# Tumor‐Derived Exosomal TAGLN2 Promotes Metastasis by Inducing Vascular Permeability and Angiogenesis via the NRP1/SEMA4D/YAP Axis

**DOI:** 10.1002/advs.202521962

**Published:** 2026-03-13

**Authors:** Shuqi Yu, Jiajia Zhuo, Xiaoquan Hong, Shihao Rao, Yafang Ye, Dandan Kang, Huifang Peng, Huiqin Zhuo

**Affiliations:** ^1^ Department of Gastrointestinal Surgery Zhongshan Hospital of Xiamen University School of Medicine Xiamen University Xiamen Fujian China; ^2^ Department of Pathology Zhongshan Hospital of Xiamen University School of Medicine Xiamen University Xiamen Fujian China; ^3^ Department of Joint Surgery and Sports Medicine Zhongshan Hospital of Xiamen University School of Medicine Xiamen University Xiamen Fujian China; ^4^ Xiamen Municipal Key Laboratory of Gastrointestinal Oncology Xiamen Fujian China; ^5^ Department of Endocrinology The First Affiliated Hospital, and College of Clinical Medicine of Henan University of Science and Technology Luoyang China

**Keywords:** angiogenesis, exosomal TAGLN2, gastric cancer, metastasis, vascular permeability

## Abstract

Tumor‐derived exosomes critically mediate metastasis, yet how specific cargoes reprogram the vasculature remains unclear. In gastric cancer (GC), we identify TAGLN2 as a key exosomal mediator. It is co‐overexpressed in GC cells and tumor‐associated endothelial cells (TECs), and its high endothelial expression correlates with lymph node metastasis and poor prognosis. Functionally, GC‐derived exosomes deliver TAGLN2 to endothelial cells (ECs), orchestrating angiogenesis, EndoMT, and the disruption of endothelial junctions. In vivo, exosomal TAGLN2 accelerated tumor growth and lung metastasis by generating abnormal, leaky vasculature and hypoxia. Mechanistically, exosomal TAGLN2 initiates a novel signaling axis: it transcriptionally upregulates *NRP1* via c‐Jun/SP1 and concurrently induces SEMA4D expression. TAGLN2 then interacts with both NRP1 and SEMA4D to nucleate a stable cytoplasmic ternary complex. This complex dually activates YAP by competitively disrupting NRP1‐YAP binding to release YAP from cytoplasmic retention, and simultaneously suppressing Hippo‐mediated degradation, operating independently of the canonical SEMA4D‐PlexinB1‐RhoA/ROCK pathway. Therapeutically, targeting the TAGLN2 axis synergized with both cisplatin and bevacizumab, potently suppressing tumor progression by impairing neovascularization and promoting vascular normalization. Clinically, exosomal TAGLN2 levels were significantly elevated in GC patient serum. Our study delineates a complete exosome‐to‐vasculature signaling axis and positions TAGLN2/NRP1/SEMA4D/YAP module as an integrated diagnostic and therapeutic target against metastatic GC.

## Graphical abstract

Gastric cancer‐derived exosomal TAGLN2 is identified as a key mediator of vascular reprogramming, with significantly elevated levels detected in patient serum. Independent of canonical SEMA4D signaling, it nucleates a cytoplasmic TAGLN2/NRP1/SEMA4D ternary complex that dually activates YAP, promoting angiogenesis, vascular dysfunction, and metastasis. Targeting this axis synergizes with chemotherapy and anti‐angiogenic therapy, positioning TAGLN2 as both a circulating biomarker and therapeutic target.

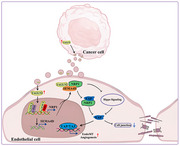

## 1 Introduction

Gastric cancer (GC) is one of the most prevalent malignancies worldwide, with distant metastasis responsible for approximately 80 % of GC‐related deaths. This clinical imperative drives the urgent need to elucidate the fundamental mechanisms governing metastatic dissemination. Accumulating evidence reveals that primary tumors can systemically precondition distant microenvironments to foster metastasis long before colonization, with tumor‐derived extracellular vesicles (EVs), particularly exosomes (TEXs, 30–150 nm), acting as pivotal long‐range messengers [1, 2]. By delivering diverse bioactive cargoes (e.g., proteins, lipids, metabolites, mRNAs, DNA, and non‐coding RNAs) to recipient cells, TEXs orchestrate multiple pro‐metastatic programs, including immunosuppression, stromal activation, metabolic adaptation, and pre‐metastatic niche formation [3, 4, 5].

Among these, the role of TEXs in remodeling the vascular endothelium has garnered increasing attention. This reprogramming, often termed vascular destabilization, primarily manifests as the induction of angiogenesis and increased vascular permeability, processes that critically facilitate metastatic spread [6, 7]. Studies have identified that specific TEX‐borne factors, such as miRNAs (e.g., miR‐25‐3p, miR‐3157‐3p, or miR‐23a‐3p), proteins (e.g., IGF2BP2), and circRNAs (e.g., Circ‐ZNF609), can promote angiogenesis and disrupt endothelial junctional proteins (e.g., ZO‐1, Occludin, Claudin 5, Claudin 1), thereby enhancing metastasis [6, 7, 8, 9, 10]. Notably, therapeutic intervention targeting such factors (e.g., using ADAM17 inhibitors to counteract exosomal ADAM17) has been shown to reduce metastasis, underscoring the translational potential of this approach [11]. Consequently, targeting TEX‐mediated vascular destabilization presents a promising therapeutic avenue. However, the molecular mechanisms orchestrating this process, particularly in GC, remain largely unexplored.

We focus on Transgelin‐2 (TAGLN2), a cytoskeletal regulator that plays a critical role in tumor progression and therapeutic resistance by integrating actin dynamics with signaling pathways and epigenetic modifications [12, 13]. TAGLN2 is overexpressed in multiple malignancies, including bladder cancer, esophageal carcinoma, colorectal cancer, GC, and glioblastoma [14, 15]. Our previous work has identified TAGLN2 as a potential biomarker of tumor‐associated endothelial cells (TECs) [16] and as a mediator that induces a resistant signature of interferon‐stimulated genes through the AKT‐YBX1 signaling axis in GC cells, which mediates IFN‐related DNA damage resistance [17]. Intriguingly, TAGLN2 has been identified in proteomic studies of exosomes, including analyses of exosomes derived from urine and colon tumor cell lines [18, 19], yet its biological functions as an exosomal cargo remain entirely unknown.

In this study, we investigate how exosomal TAGLN2 acts as a crucial link between GC cells and endothelial cells (ECs) to drive vascular destabilization to accelerate metastasis. We demonstrate that GC‐derived exosomes efficiently deliver functional TAGLN2 to ECs, where it orchestrates angiogenesis, EndoMT, and the disruption of endothelial integrity. Mechanistically, we identify the NRP1/SEMA4D/YAP signaling axis as the mechanistic core through which exosomal TAGLN2 reprograms the endothelium. To bridge these mechanistic insights with clinical relevance, we evaluate the levels of exosomal TAGLN2 in GC patient serum, and assess the therapeutic potential of targeting this axis, including its synergy with cisplatin chemotherapy and the anti‐VEGF agent bevacizumab. Together, our findings establish exosomal TAGLN2 as a master regulator of vascular remodeling in GC metastasis, and validate it as a compelling biomarker and therapeutic target.

## 2 Results

### 2.1 Synergistic Upregulation of TAGLN2 in Gastric Cancer Cells and Tumor Endothelium

**FIGURE 1:**
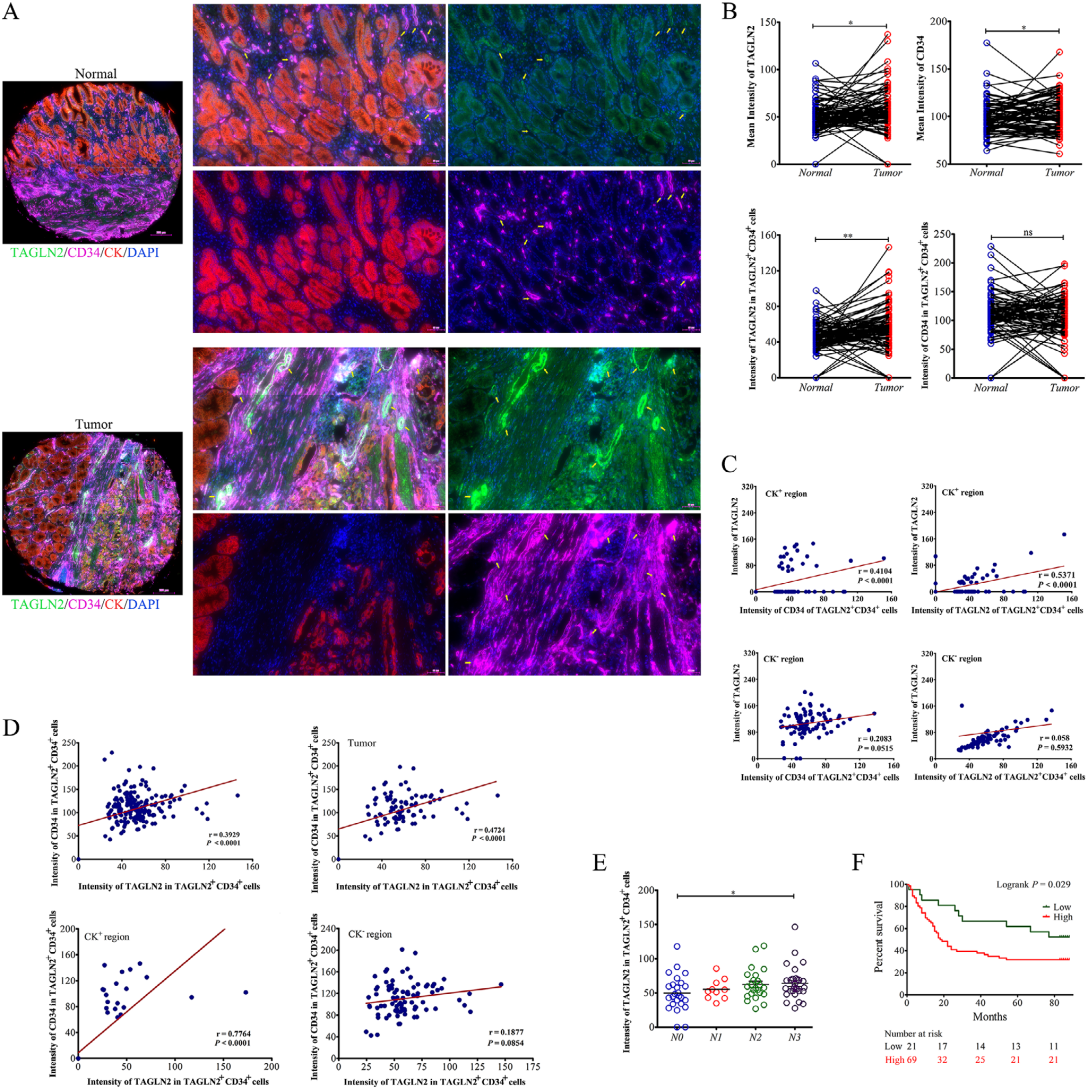
Aberrantly increased expression of TAGLN2 in GC microenvironment. (A) Multiplex immunofluorescence analysis of 90 matched GC and adjacent normal tissues using antibodies against TAGLN2 (Opal 520, green), CK (Opal 570, red), CD34 (Opal 690, purple), and DAPI (blue). Scale bars: 200 µm (overview), 50 µm (insets). (B) Comparative analysis of TAGLN2 and CD34 expression in tumor vs. matched normal tissues and endothelial cells. (C) Correlation between tumor cell‐derived TAGLN2 and endothelial expression of TAGLN2 or CD34 (intensity in TAGLN2^+^CD34^+^ cells) within CK^+^ and CK^−^ regions. (D) Correlation between endothelial TAGLN2 and CD34 expression across all tissues, total tumor, and CK^+^ or CK^−^ tumor regions. (E) High endothelial TAGLN2 intensity is significantly associated with advanced N stage. (F) Kaplan–Meier survival curve showing that elevated endothelial TAGLN2 correlates with reduced overall survival. ns, not significant; ^*^ *p*< 0.05, ^**^ *p*< 0.01.

A multiplex immunofluorescence panel targeting TAGLN2, CD34 (endothelial cell marker), CK (epithelial cell marker), and DAPI was utilized to analyze 90 tumor tissues and paired normal tissues from GC patients (Figure 1A). The expression levels of both TAGLN2 and CD34 (DAPI^+^ cells) were significantly elevated in tumor tissues compared to paracancerous tissues (Figure 1B). Within tumor‐associated endothelial cells (TAGLN2^+^CD34^+^), TAGLN2 expression was markedly higher than that in matched normal tissues, whereas CD34 levels showed no significant difference. A strong positive correlation was observed between tumor cell‐derived TAGLN2 and endothelial CD34 expression (*r* = 0.4104, *p*< 0.0001), as well as between tumor cell‐derived TAGLN2 and endothelial TAGLN2 expression (*r* = 0.5371, *p*< 0.0001). These correlations were specific to CK^+^ tumor regions, with no significant correlation in CK^−^ regions (Figure 1C). Furthermore, TAGLN2 and CD34 expression within ECs (TAGLN2^+^CD34^+^) were positively correlated across all tissues (*r* = 0.3929, *p*< 0.0001). This correlation was more pronounced within tumor tissues (*r* = 0.4724, *p*< 0.0001), and was strongest in CK^+^ regions (*r* = 0.7764, *p*< 0.0001), with no significant correlation in CK^−^ regions (Figure 1D). High endothelial TAGLN2 intensity was significantly associated with the presence of lymphatic invasion, advanced N stage, and reduced overall survival in GC patients (Figure 1E,F). No significant links were found with other clinical indicators, as detailed in Table S1.

Pan‐cancer analysis revealed consistently elevated *TAGLN2* mRNA expression across multiple malignancies, including STAD, COAD, LUAD, BLCA, and BRCA (Figure S1A). Analysis of 27 paired samples from TCGA STAD (Figure S1B) and 40 pairs from our institutional gastrointestinal cancer biobank (Figure S1C) confirmed *TAGLN2* overexpression in gastric tumor tissues, with higher levels predicting worse prognosis and reduced overall survival (Figure S1D). Functional enrichment analysis indicated that *TAGLN2* co‐expressed genes were primarily involved in cadherin binding, cell adhesion mediator, and disulfide oxidoreductase activity, while negatively correlated genes were mainly involved in actin binding, metal ion transmembrane transporter activity, and microtubule binding (Figure S1E).

### 2.2 TAGLN2 Induces Endothelial Hyperpermeability and Angiogenesis

**FIGURE 2:**
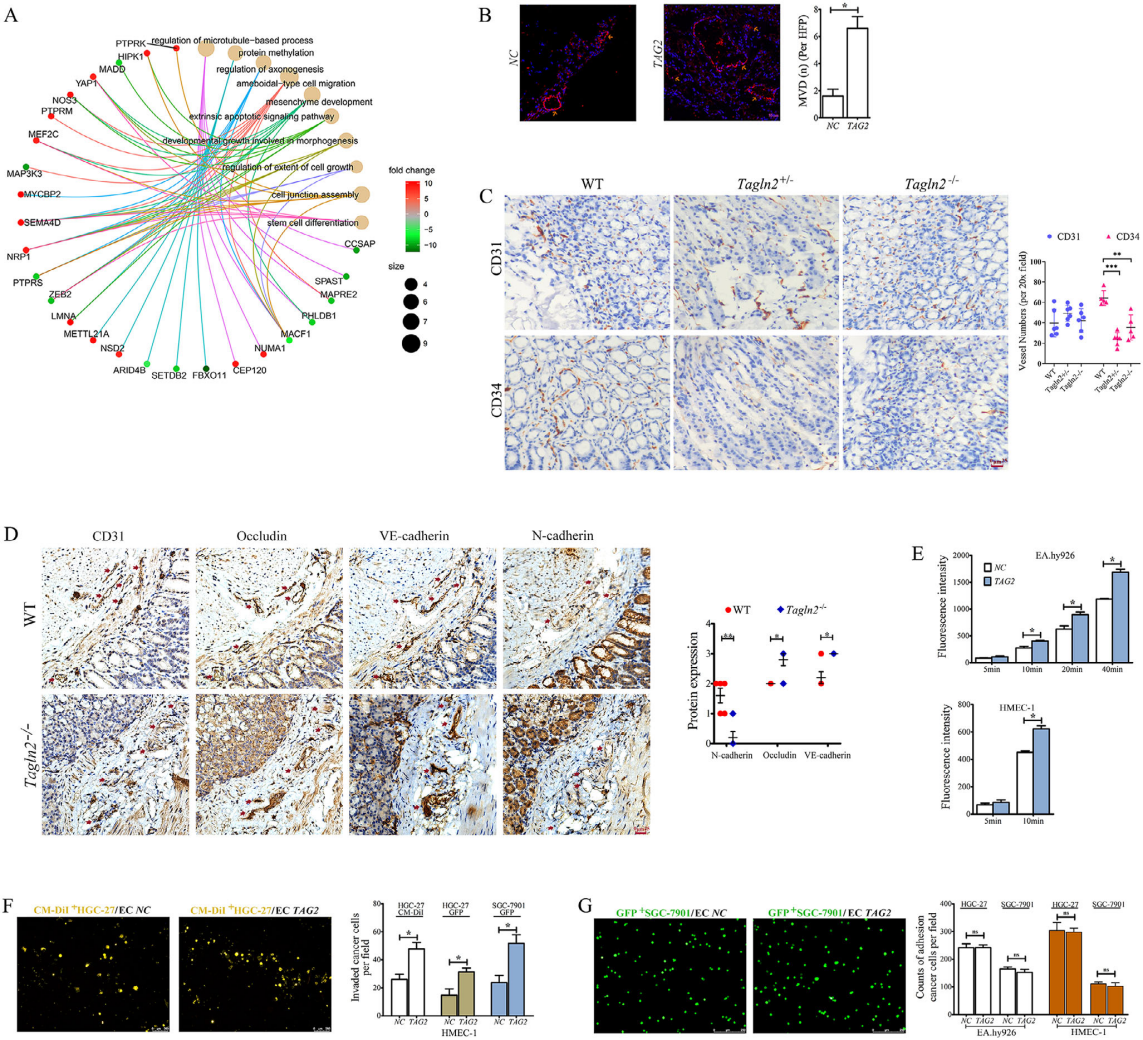
TAGLN2 induces endothelial hyperpermeability and angiogenesis. (A) RNA‐seq transcriptomic profiling of primary HUVECs overexpressing *TAGLN2* vs. mock controls. The top 10 enriched GO terms among the significant DEGs are shown. (B) Matrigel plug assay. Plugs containing EA.hy926 cells overexpressing TAGLN2 (*TAG2*) or mock cells were subcutaneously implanted into BALB/c nude mice (*n* = 4). After 10 days, plugs were sectioned and immunostained for vWF (red) with DAPI counterstain (blue). (C, D) Immunostaining of gastric tissues from wild‐type (WT), *Tagln2* heterozygous (*Tagln2* ^+/−^) and knockout (*Tagln2* ^−/−^) mice for endothelial markers (CD34, CD31) and junctional proteins (Occludin, VE‐cadherin, N‐cadherin within CD31^+^ endothelial layers). (E) Endothelial permeability in EA.hy926 and HMEC‐1 cells following TAGLN2 overexpression, measured by Rhodamine‐dextran (70 kDa, 20 mg/mL) efflux. (F, G) Representative images (left) and quantification (right) of transendothelial invasion (F) and tumor‐endothelium adhesion (G) assays. GC cells (CM‐DiI‐labeled HGC‐27, HGC‐27/GFP, or SGC‐7901/GFP) were co‐cultured with *TAGLN2*‐overexpressing endothelial cells. Data are presented as mean ± SD. ns, not significant; ^*^ *p*< 0.05, ^**^ *p*< 0.01, ^***^ *p*< 0.001.

Transcriptomic profiling of *TAGLN2*‐overexpressing vs. control primary HUVECs by RNA‐seq identified 86 differentially expressed genes. The TOP 10 enriched Gene Ontology (GO) terms primarily included ameboidal‐type cell migration, cell junction assembly, developmental growth involved in morphogenesis, apoptotic signal, cell growth, and microtubule organization (Figure 2A). Key implicated genes included *NRP1*, *SEMA4D*, *ZEB2*, *MACF1*, *PTPRS*, *MEF2C* and *YAP*, among which *NRP1*, *SEMA4D*, *MEF2C* and *YAP* exhibited more than 8.5 fold (log_2_^FC^) upregulation.

In vivo, matrigel plugs containing *TAGLN2*‐overexpressing ECs exhibited a markedly increased density of neovessels, as indicated by vWF staining (Figure 2B). Immunostaining of gastric tissues from *Tagln2* heterozygous (*Tagln2* ^+/−^) and knockout (*Tagln2* ^−/−^) mice revealed a significant decrease in CD34 expression (neovascularization marker), while CD31 expression (a mature vasculature marker) showed no significant change (Figure 2C).

Integrating RNA‐seq profiling, morphological observations, and GO enrichment analyses, our findings indicated that TAGLN2 plays a critical role in regulating endothelial cell–cell junctions and endothelial‐mesenchymal transition (EndoMT), processes closely linked to endothelial monolayer permeability. Immunostaining of GC tissues from *Tagln2* ^−/−^ mice, using CD31 to identify endothelial layers, revealed significantly elevated expression of the junctional proteins Occludin and VE‐cadherin, along with reduced levels of the mesenchymal marker N‐cadherin, compared with wild‐type controls (Figure 2D).

Functionally, overexpression of TAGLN2 in EA.hy926 or HMEC‐1 endothelial monolayers significantly increased permeability to Rhodamin‐dextran (Figure 2E). Consistent with this barrier disruption, we observed enhanced transendothelial invasion of labeled tumor cells (CM‐DiI‐labeled HGC‐27, HGC‐27/GFP, or SGC‐7901/GFP) through *TAGLN2*‐overexpressing endothelial monolayers (Figure 2F), while their adhesion to the endothelium remained unchanged (Figure 2G), as shown by representative images and corresponding quantification.

### 2.3 NRP1/SEMA4D Axis Mediates TAGLN2‐Driven Vascular Dysfunction

**FIGURE 3:**
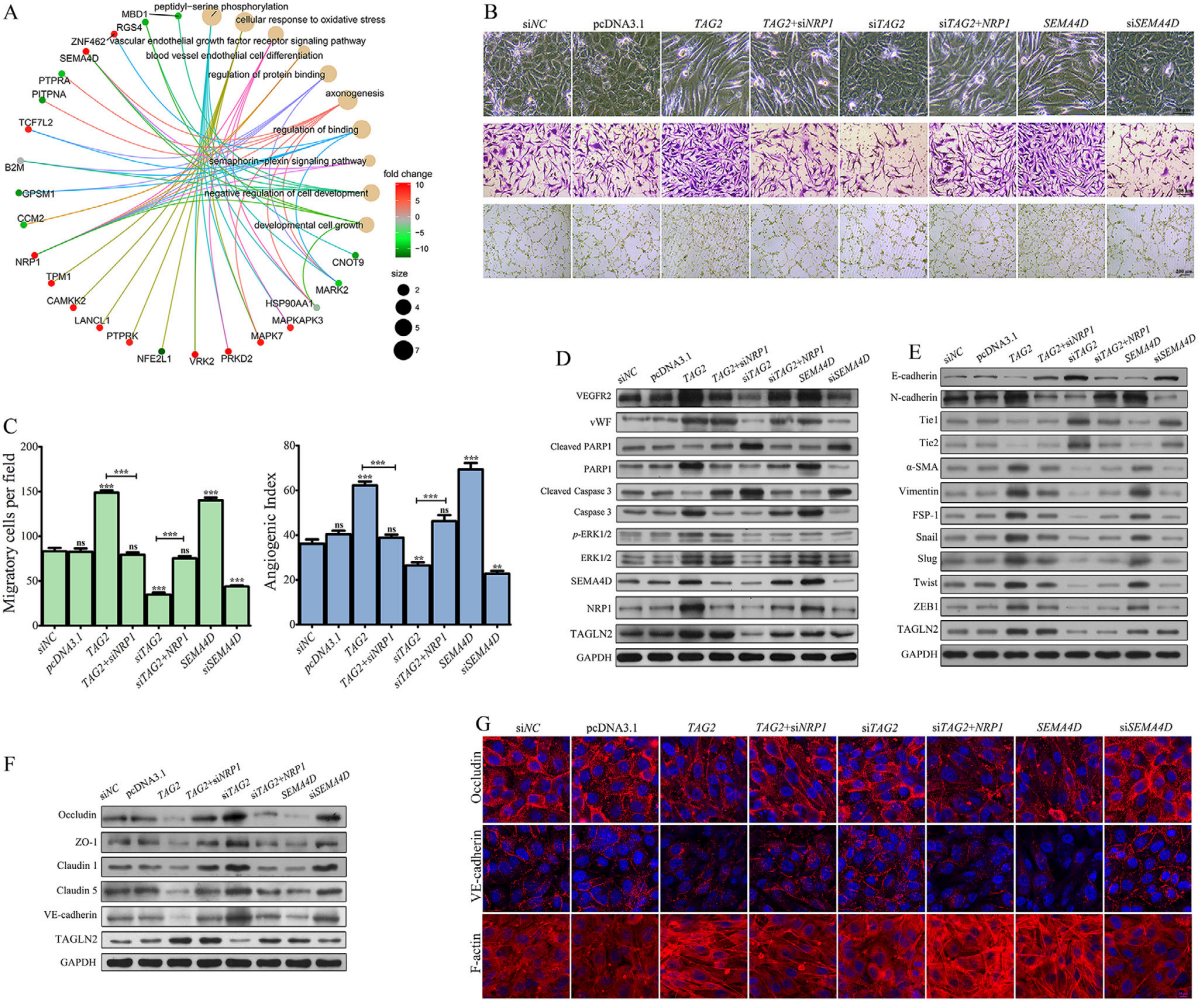
TAGLN2 promotes vascular dysfunction through the NRP1/SEMA4D axis. (A) RNA‐Seq transcriptomic profiling of *NRP1*‐overexpressing vs. mock‐treated primary HUVECs. The top 10 enriched GO terms among significant DEGs are shown. (B) Cell migration and (C) capillary‐like tube formation were assessed following the indicated genetic manipulations (*TAGLN2* or *SEMA4D* overexpression/knockdown, with or without concomitant modulation of *NRP1*). (D) Expression of core axis components (NRP1, SEMA4D), angiogenesis markers (VEGFR2, vWF), MAPK pathway markers (ERK1/2, *p*‐ERK1/2), and apoptosis markers (Cleaved PARP1 and Caspase 3). (E) Expression of EndoMT‐related markers: endothelial markers (E‐cadherin, Tie 1/2), mesenchymal markers (N‐cadherin, α‐SMA, FSP‐1, Vimentin), and EMT‐transcription factors (Snail, Slug, Twist, ZEB1). (F) Expression of junctional proteins (VE‐cadherin, Occludin, ZO‐1, Claudin 1, Claudin 5). (G) Immunofluorescence staining for Occludin, VE‐cadherin, and F‐actin (Phalloidin) to visualize junctional and cytoskeletal organization. (B–G) The EA.hy926 cell line was used in all experiments. Data are presented as mean ± SD. Significance vs. si*NC* control: ns, not significant; ^*^ *p*< 0.05, ^**^ *p*< 0.01, ^***^ *p*< 0.001. Lines with symbols denote pairwise comparisons.

Given the established role of NRP1 as a co‐receptor for VEGF in angiogenesis, along with its striking 9 fold (log_2_^FC^) upregulation and its central representation in the TOP 10 enriched GO terms, we reasoned that *NRP1* overexpression is a pivotal downstream event of TAGLN2. To systematically uncover the transcriptional network, we performed RNA‐seq on *NRP1*‐overexpressing HUVECs, identifying 94 differentially expressed genes (Figure 3A). Functional enrichment revealed pathways critical for vascular biology, including axonogenesis, endothelial cell differentiation, response to oxidative stress, developmental cell growth, semaphorin‐plexin signaling pathway, and vascular endothelial growth factor receptor signaling pathway. Notably, key genes such as *SEMA4D*, *B2M*, *MAPK7*, *HSP90AA1*, and *MARK2* were significantly upregulated. The most striking finding was that both *TAGLN2* and *NRP1* overexpression induced a greater than 10 fold (log_2_^FC^) increase in *SEMA4D* expression. These findings collectively define a coherent TAGLN2‐NRP1‐SEMA4D transcriptional axis.

Overexpression of TAGLN2 or SEMA4D individually induced a spindle‐like morphological transition in ECs, along with significantly enhanced cell migration and capillary‐like tube formation, whereas silencing either gene reversed these phenotypic and functional changes, and these effects were reversible upon respective gene silencing (Figure 3B,C). Notably, both the morphological and the pro‐angiogenic effects driven by TAGLN2 were largely abolished upon *NRP1* knockdown. Moreover, re‐expression of NRP1 effectively rescued the impairments caused by TAGLN2 knockdown. At the molecular level, TAGLN2 overexpression increased protein levels of NRP1, SEMA4D (neither NRP1 nor SEMA4D modulation affected TAGLN2 expression), angiogenesis markers (VEGFR2 and vWF), and activated the MAPK/ERK pathway (ERK1/2 and *p*‐ERK1/2), while decreasing apoptosis markers (cleaved PARP1 and Caspase 3). Conversely, TAGLN2 knockdown yielded the opposite effect. *NRP1* knockdown prevented TAGLN2‐induced upregulation of SEMA4D, VEGFR2, and vWF upregulation and reversed the suppression of apoptosis markers (Figure 3D).

In the same experimental settings, the TAGLN2/NRP1/SEMA4D axis orchestrated a comprehensive EndoMT program. It drove the downregulation of endothelial markers (E‐cadherin and Tie1/2) and the upregulation of mesenchymal markers (N‐cadherin, α‐SMA, FSP‐1, Vimentin), along with the core EMT‐transcription factors Snail, Slug, Twist, and ZEB1 (Figure 3E). As observed for the angiogenic markers, NRP1 was required for TAGLN2 to induce this EndoMT signature, and NRP1 overexpression could restore it upon TAGLN2 knockdown. Concomitantly, this axis disrupted endothelial barrier integrity by downregulating key adherens junction (VE‐cadherin) and tight junction (Occludin, ZO‐1, Claudin 1, Claudin 5) proteins, and the effect also depended on NRP1 (Figure 3F). Immunofluorescence analysis confirmed the loss of VE‐cadherin and Occludin from cell junctions upon axis activation (Figure 3G). As an actin‐binding protein, TAGLN2 plays a critical role in F‐actin stabilization. Activation of this axis significantly increased actin filament density, enhanced F‐actin polymerization, and promoted thick actin stress fiber formation. Collectively, these findings establish that the NRP1/SEMA4D axis is the central mechanism through which TAGLN2 reprograms ECs, coordinating pro‐angiogenic, pro‐migratory, mesenchymal‐transitional, barrier‐disrupting, and cytoskeletal changes.

### 2.4 TAGLN2 Upregulates *NRP1* Expression and Activates YAP via the NRP1/SEMA4D Axis

**FIGURE 4:**
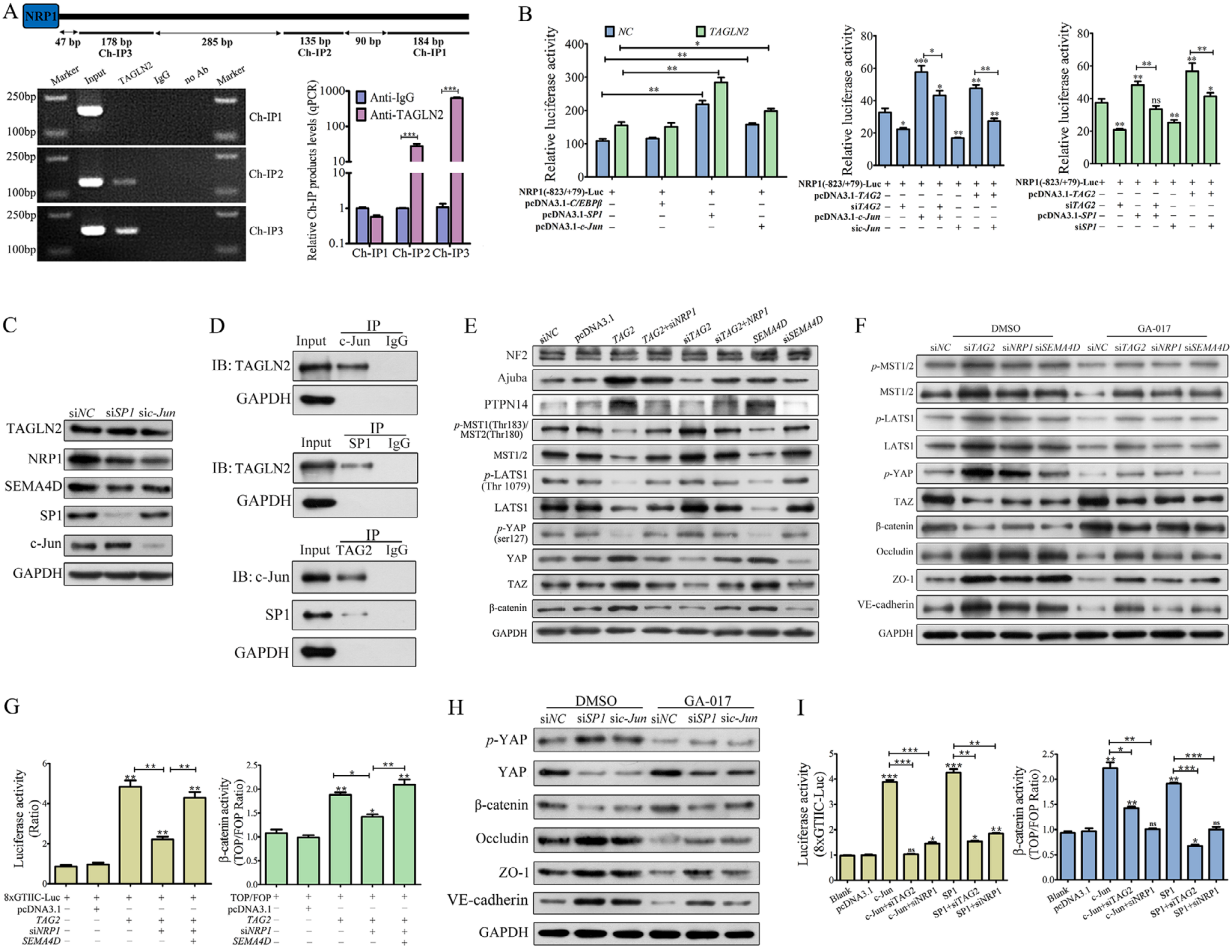
TAGLN2 upregulates NRP1 transcription and activates YAP through the NRP1/SEMA4D axis. (A) ChIP‐qPCR analysis of endogenous TAGLN2 binding to the *NRP1* promoter region. (B) Luciferase reporter assays evaluating the transcriptional activity of the *NRP1* promoter (−823/+79) in response to C/EBPβ, SP1, and c‐Jun, with or without *TAGLN2* overexpression. (C) Protein levels of TAGLN2, NRP1, and SEMA4D following *SP1* or *c‐Jun* knockdown. (D) Co‐IP assays assessing endogenous interactions between TAGLN2 and SP1 or c‐Jun. (E) Levels of core Hippo pathway kinases following genetic perturbation of the TAGLN2/NRP1/SEMA4D axis. (F) Treatment with the LATS1/2 inhibitor GA‐017 (10 µM, 3 h) reversed Hippo signaling activation and junctional protein upregulation induced by knockdown of *TAGLN2*, *NRP1*, or *SEMA4D*. (G) Transcriptional activity of TEAD (8× GTIIC‐Luc reporter), and β‐catenin/TCF (TOPFlash/FOPFlash reporter system) following axis modulation. (H, I) Effects of *SP1* or *c‐Jun* knockdown on Hippo pathway activity, junctional protein expression, and transcriptional activity of TEAD or β‐catenin/TCF reporters. The EA.hy926 cell line was used in all experiments. Data are presented as mean ± SD. Significance vs. respective control: ns, not significant; ^*^ *p*< 0.05, ^**^ *p*< 0.01, ^***^ *p*< 0.001. Lines with symbols denote pairwise comparisons.

ChIP followed by qPCR and agarose gel electrophoresis revealed significant enrichment (over 600 fold) of TAGLN2 at the *NRP1* promoter region (−225 to −1 bp) compared to the negative control (Figure 4A). Since *NRP1* expression is known to be cooperatively regulated by transcription factors such as AP‐1, SP1, and C/EBPβ [20], we evaluated three candidates using a luciferase reporter assay. Co‐transfection of the *NRP1* promoter reporter with c‐Jun (the primary component of AP‐1), or SP1, but not C/EBPβ, significantly enhanced luciferase activity, with a more pronounced effect in *TAGLN2*‐overexpressing ECs (Figure 4B). Gain‐ or loss‐of‐function experiments confirmed that TAGLN2, c‐Jun, and SP1 positively regulated *NRP1* promoter activity. Crucially, *TAGLN2* knockdown attenuated the enhancer effects of c‐Jun or SP1, while depletion of c‐Jun or SP1 abolished TAGLN2‐induced *NRP1* promoter activation. Furthermore, knockdown of c‐Jun or SP1 reduced protein levels of NPR1 and SEMA4D without affecting TAGLN2 levels (Figure 4C). Co‐IP confirmed direct physical interactions between endogenous TAGLN2 and both c‐Jun and SP1 (Figure 4D). Together, these results demonstrate that TAGLN2 transcriptionally upregulates *NRP1* expression by facilitating the recruitment of c‐Jun and SP1 to its promoter.

As RNA‐seq identified *YAP* as a significantly upregulated gene upon TAGLN2 overexpression, and YAP is known to play multifaceted roles in angiogenesis and endothelial junctional stability, we examined its regulation through the Hippo pathway core kinase cascade. Overexpression of TAGLN2 or SEMA4D markedly reduced protein levels of MST1/2, *p*‐MST1(Thr183)/MST2(Thr180), LATS1, *p*‐LATS1(Thr1079) and *p*‐YAP(Ser127) (Figure 4E). Concurrently, levels of Ajuba (a positive regulator of YAP that inhibits LATS1/2), PTPN14, YAP, TAZ, and β‐catenin (released from the destruction complex) were significantly increased, whereas the upstream regulator NF2 remained unchanged. Knockdown of TAGLN2 or SEMA4D reversed these effects, and NRP1 knockdown abrogated TAGLN2‐mediated suppression of Hippo signaling.

Treatment with the Hippo pathway inhibitor GA‐017 (targeting LATS1/2) decreased the levels of MST1/2, *p*‐MST1/MST2, LATS1, *p*‐LATS1, and *p*‐YAP, and increased the levels of TAZ and β‐catenin in *TAGLN2*, *NRP1*, *SEMA4D*‐knockdown EA.hy926 cells (Figure 4F). Furthermore, GA‐017 remarkably reversed the upregulation of junctional proteins (Occludin, ZO‐1, and VE‐cadherin) induced by knockdown of *TAGLN2*, *NRP1*, or *SEMA4D*, confirming that Hippo pathway inactivation is central to the axis‐mediated barrier disruption.

To functionally assess YAP/TEAD transcriptional output, we used an 8× GTIIC‐Luc reporter construct containing eight synthetic TEAD‐binding sites. TAGLN2 overexpression enhanced TEAD‐dependent transcriptional activity by over 6 fold, an effect abolished by *NRP1* knockdown but restored by SEMA4D supplementation (Figure 4G). A similar pattern was observed for β‐catenin/TCF‐dependent transcription using the TOPFlash/FOPFlash reporter system. As anticipated, GA‐017 treatment reduced *p*‐YAP and junction protein levels (Occludin, ZO‐1, VE‐cadherin), while elevating YAP and β‐catenin in *SP1*‐ or *c‐Jun*‐deficient cells (Figure 4H). Finally, SP1 or c‐Jun overexpression significantly enhanced 8× GTIIC‐Luc reporter activity, which was nearly completely abolished by TAGLN2 or NRP1 knockdown (Figure 4I). A concordant pattern was observed in TOPFlash/FOPFlash reporter assays. These results establish YAP as a critical downstream effector of the TAGLN2/NRP1/SEMA4D axis, essential for mediating its impact on endothelial modulation.

### 2.5 Cytoplasmic TAGLN2/NRP1/SEMA4D Ternary Complex Activates YAP via a PlexinB1‐RhoA/ROCK Independent Mechanism

**FIGURE 5:**
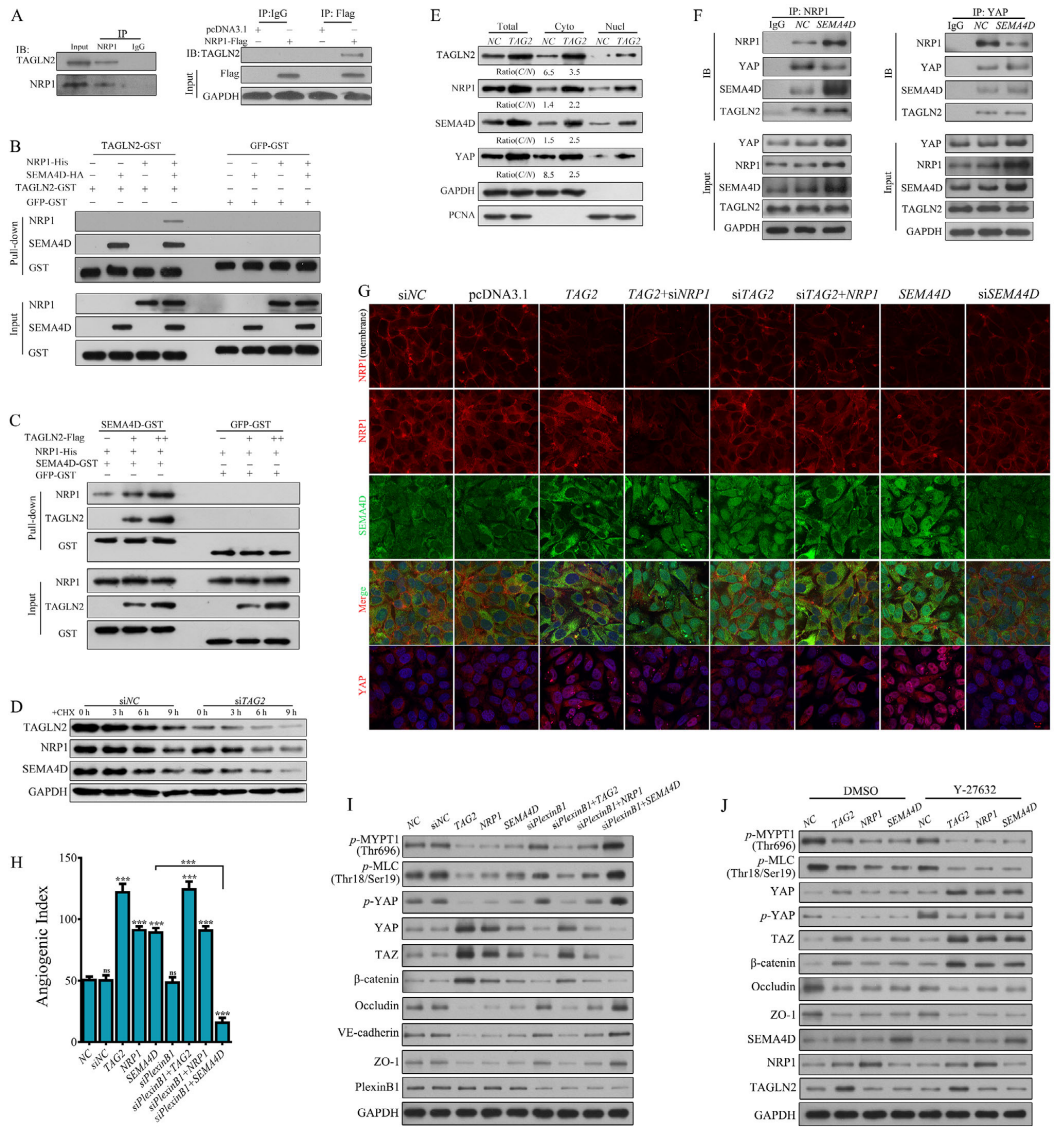
TAGLN2 stabilizes the NRP1‐SEMA4D complex to activate YAP independently of the canonical PlexinB1‐RhoA/ROCK pathway. (A) Endogenous interaction between TAGLN2 and NRP1 was confirmed by Co‐IP using antibodies against NRP1 or the Flag tag (for exogenously expressed Flag‐NRP1). (B, C) In vitro pull‐down assays with purified recombinant proteins. Increasing amounts of TAGLN2 (100–200 µg) enhanced the direct interaction between NRP1 and SEMA4D. (D) Cycloheximide (CHX, 25 µg/mL) chase assay evaluating the protein stability of NRP1 and SEMA4D with or without *TAGLN2* knockdown. (E) Subcellular fractionation and western blot analysis of TAGLN2, NRP1, SEMA4D, and YAP in EA.hy926 cells with or without *TAGLN2* overexpression. (F) Competitive binding assay showing SEMA4D disrupts the cytoplasmic NRP1‐YAP interaction by Co‐IP. (G) Immunofluorescence analysis of subcellular localization for NRP1 (with or without 0.3 % Triton X‐100 pre‐permeabilization), SEMA4D, and YAP following axis modulation. Scale bar: 10 µm. (H) Quantification of capillary tube formation in EA.hy926 cells overexpressing *TAGLN2*, *NRP1*, or *SEMA4D*, with or without *PlexinB1* knockdown. (I) Western blot analysis of key signaling molecules involved in YAP activation, ROCK suppression (*p*‐MYPT1, *p*‐MLC), and junction disassembly in EA.hy926 cells overexpressing *TAGLN2*, *NRP1*, or *SEMA4D*, with or without *PlexinB1* knockdown. (J) Western blot analysis of indicated signaling molecules in EA.hy926 cells treated with the ROCK inhibitor Y‐27632 (10 µM, 4 h), with or without axis activation. Data are presented as mean ± SD. Significance vs. *NC* control: ns, not significant; ^*^ *p*< 0.05, ^**^ *p*< 0.01, ^***^ *p*< 0.001. Lines with symbols denote pairwise comparisons.

Co‐IP confirmed the endogenous interaction between TAGLN2 and NRP1 (Figure 5A). GST pull‐down assays further revealed that the binding of GST‐TAGLN2 to His‐NRP1 was dependent on the presence of SEMA4D (Figure 5B). Moreover, increasing amounts of recombinant TAGLN2 (100–200 µg) enhanced the interaction between GST‐SEMA4D and NRP1 by 2.3–4.6 fold (Figure 5C), indicating that TAGLN2 facilitates ternary complex formation. To assess the effect of TAGLN2 on protein stability, a cycloheximide (CHX, 25 µg/mL) chase assay was performed. Knockdown of TAGLN2 accelerates the degradation of both NRP1 and SEMA4D, reducing their half‐lives from approximately 7.7–4.8 h and from 6.0 to 4.6 h, respectively, while the stability of SP1 remained unaffected (Figure 5D, Figure S2A).

Previous studies indicate that cytoplasmic retention of YAP supports endothelial barrier integrity, while its nuclear translocation disrupts cell–cell junctions [21]. Subcellular fractionation revealed that TAGLN2 overexpression increased the cytoplasmic accumulation of NRP1 and SEMA4D but markedly reduced the cytoplasmic‐to‐nuclear ratio of YAP (from 8.5 to 2.5), indicating enhanced YAP nuclear translocation (Figure 5E). Given that NRP1 can bind and retain YAP in the cytoplasm [22], we investigated the impact of the ternary complex on this interaction. Co‐IP assays showed that SEAM4D overexpression markedly reduced the binding between NRP1 and YAP, while simultaneously enhancing the interaction among NRP1, TAGLN2, and SEMA4D. Reciprocal Co‐IP using an anti‐YAP antibody confirmed that SEMA4D profoundly diminished NRP1‐YAP association (Figure 5F). These results demonstrate that SEMA4D competitively binds to NRP1 in the cytoplasm, thereby displacing YAP from the NRP1‐YAP complex.

Immunofluorescence under selective permeabilization conditions showed that overexpression of TAGLN2 or SEMA4D redistributed NRP1 from the membrane to the cytoplasm. Conversely, their knockdown reduced cytoplasmic NRP1 without affecting its membrane localization. SEMA4D, which is normally distributed in both the cytoplasm/membrane and nucleus, translocated entirely to the cytoplasm and co‐localized with NRP1 when NRP1 levels were elevated (e.g., in *TAG2*‐ or si*TAG2*+*NRP1* groups), concomitant with YAP upregulation and nuclear accumulation. In contrast, when NRP1 levels were low (e.g., si*TAG2*‐ or *TAG2*+si*NRP1* groups), SEMA4D localized exclusively to the nucleus, and YAP expression and nuclear translocation were suppressed (Figure 5G).

SEMA4D is a membrane‐anchored protein that can be cleaved to release a soluble fragment (∼120 kDa, sSEMA4D), which classically signals through its receptor PlexinB1 to disrupt endothelial junctions [23]. To determine whether the TAGLN2/NRP1/SEMA4D axis operates through this canonical paracrine mechanism, we first examined sSEMA4D secretion. ELISA analysis revealed that sSEMA4D levels increased significantly only when SEMA4D was overexpressed alone in either GC or ECs. In stark contrast, modulation of TAGLN2, NRP1, SP1, or c‐Jun alone or in combination did not induce detectable sSEMA4D release (Figure S2B). This indicates that the axis does not rely on generating the soluble ligand to exert its function.

We next directly interrogated the requirement for the canonical receptor PlexinB1. In capillary tube formation assays, the potent pro‐angiogenic effects driven by TAGLN2 or NRP1 overexpression were largely preserved upon PlexinB1 knockdown (si*PlexinB1*), with no statistically significant attenuation compared to their effects in receptor‐sufficient cells (Figure 5H; Figure S2C). In stark contrast, the enhanced tube formation induced by SEMA4D overexpression alone was completely abolished upon PlexinB1 knockdown (si*PlexinB1*+*SEMA4D*); instead, this combination significantly inhibited tubulogenesis, reducing network formation to a level far below the control.

Consistent with the functional data, molecular profiling confirmed this dichotomy. The key signaling events driven by the axis, including YAP activation (decreased *p*‐YAP, increased YAP, TAZ, β‐catenin), suppression of ROCK activity (decreased *p*‐MYPT1/*p*‐MLC), and disassembly of endothelial junctions (downregulation of Occludin, VE‐cadherin, ZO‐1), remained strongly sustained in PlexinB1‐deficient cells overexpressing TAGLN2, and were substantially maintained (though moderately attenuated) in cells overexpressing NRP1 alone (Figure 5I). Conversely, all effects induced by SEMA4D overexpression alone were completely reversed upon PlexinB1 knockdown, with *p*‐MYPT1/*p*‐MLC, *p*‐YAP, and junctional protein levels rising significantly above baseline, genetically proving that the ternary complex engages a signaling route independent of the classic SEMA4D receptor.

To further dissect the relationship with the canonical downstream pathway, we employed the ROCK‐specific inhibitor Y‐27632. As expected, inhibition of ROCK suppressed its own activity (reduced *p*‐MYPT1/*p*‐MLC2). Notably, activation of the TAGLN2/NRP1/SEMA4D axis also strongly suppressed ROCK activity even in the absence of the inhibitor. Combination of Y‐27632 with axis activation further suppressed ROCK activity and disrupted cell junctions (e.g., ZO‐1 loss), while synergistically amplifying YAP activation (Figure 5J). This demonstrates that the axis does not require ROCK activity to activate YAP; rather, it acts in parallel to suppress ROCK, and the two interventions converge to maximally de‐repress YAP. Collectively, these data from three independent angles establish that the cytoplasmic TAGLN2/NRP1/SEMA4D complex activates YAP through a mechanism that bypasses both the canonical receptor PlexinB1 and its classic effector RhoA/ROCK.

### 2.6 Exosomal TAGLN2 Promotes Angiogenesis and Endothelial Dysfunction via the NRP1/SEMA4D/YAP Axis

Gain‐ and loss‐of‐function analyses in GC cell lines confirmed the oncogenic role of TAGLN2, promoting proliferation, migration, invasion, clone formation, and cell cycle progression, while inhibiting apoptosis (Figure S3). GO analysis suggested an association of TAGLN2 with EVs/exosomes. The strong correlation between tumor cell‐derived and endothelial TAGLN2 expression within CK^+^ tumor regions (Figure 1C), coupled with our prior observation of elevated TAGLN2 protein but not mRNA in tumor ECs, supports the hypothesis of intercellular TAGLN2 transfer via exosomes.

**FIGURE 6:**
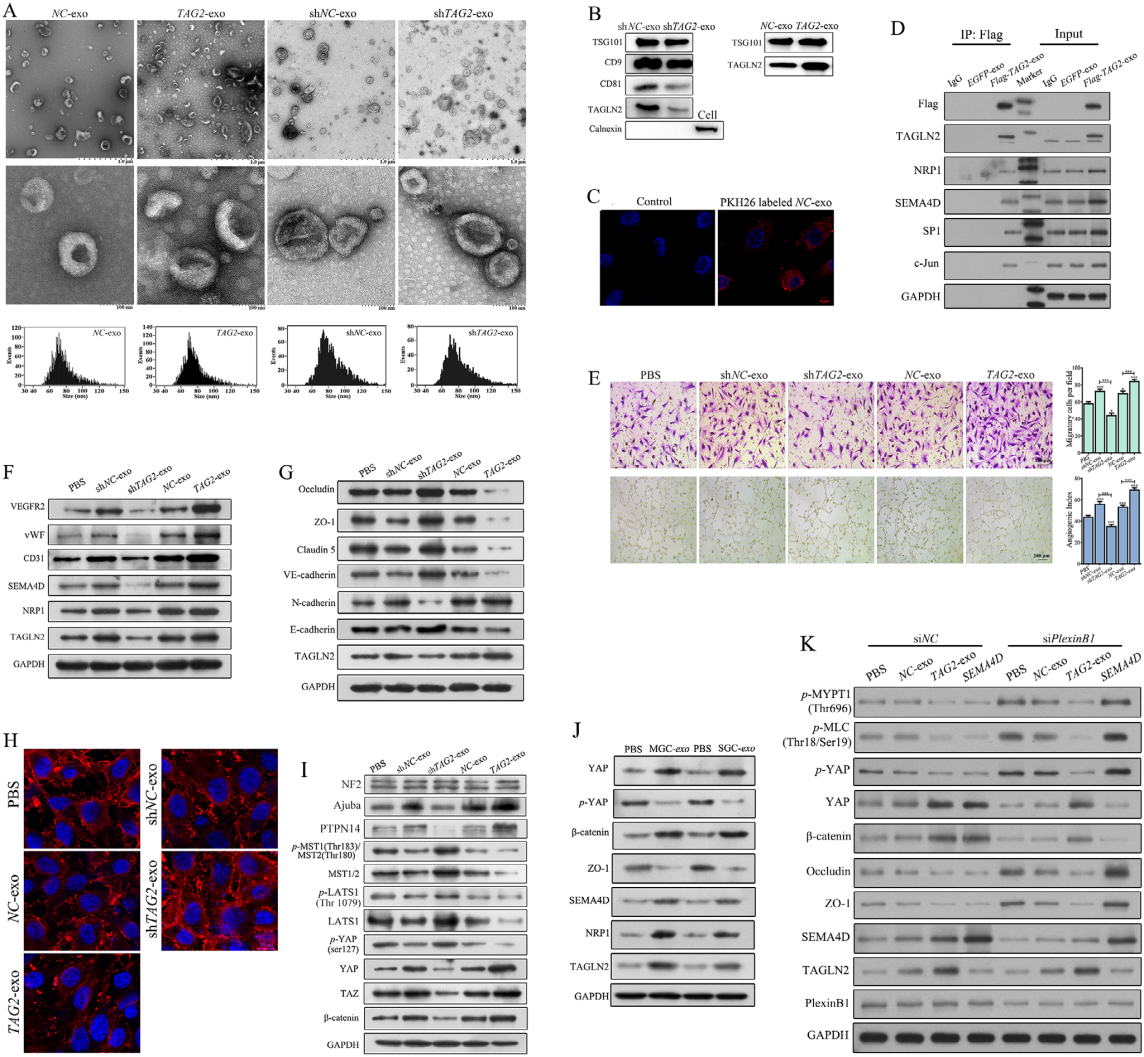
GC‐derived exosomal TAGLN2 promotes angiogenesis and endothelial dysfunction through activation of the NRP1/SEMA4D/YAP axis. (A) Representative TEM images and NTA of exosomes derived from BGC‐823 cells with *TAGLN2* overexpression (*TAG2*‐exo), knockdown (sh*TAG2*‐exo), or respective controls (*NC*‐exo or sh*NC*‐exo). Scale bar, 100 nm. (B) Western blot analysis of exosomal markers (TSG101, CD9, CD81), the negative marker Calnexin, and TAGLN2. (C) Confocal microscopy showing intracellular uptake of PKH26‐labeled exosomes (red) by EA.hy926 cells. (D) Direct biochemical evidence of exosomal TAGLN2 interaction. Co‐IP using anti‐Flag antibody on lysates from ECs co‐cultured with exosomes derived from BGC‐823 cells stably expressing 3×Flag‐TAGLN2 or EGFP (control). Blots show Flag‐TAGLN2 and co‐precipitated endogenous NRP1, SEMA4D, SP1, and c‐Jun. (E) Representative images and quantification of cell migration and tube formation in ECs treated with 5 µg/mL of *NC*‐exo, sh*NC*‐exo, sh*TAG2*‐exo, and *TAG2*‐exo for 24 h. (F, G) Western blot analysis of pro‐angiogenic factors, junctional proteins, and EndoMT markers in ECs following exosome treatment. (H) Representative immunofluorescence images showing the Occludin localization at cell–cell contacts in ECs following exosome treatment. (I) Western blot analysis of core Hippo pathway components in ECs treated with exosomes. (J) Western blot validation of the axis using exosomes derived from other GC cell lines (MGC‐803 and SGC‐7901), assessing levels of TAGLN2, NRP1, SEMA4D, Hippo pathway components, and junctional proteins. (K) Western blot analysis of key signaling molecules in si*NC* and si*PlexinB1* ECs treated with PBS, *NC*‐exo, or *TAG2*‐exo. Data are presented as mean ± SD. Significance vs. PBS control: ns, not significant; ^*^ *p*< 0.05, ^**^ *p*< 0.01, ^***^ *p*< 0.001. Lines with symbols denote pairwise comparisons.

Exosomes were isolated from the BGC‐823 cells with *TAGLN2* overexpression (*TAG2*‐exo), knockdown (sh*TAG2*‐exo), or respective controls (*NC*‐exo or sh*NC*‐exo). They exhibited typical cup‐shaped morphology and an average size of ∼78 nm (Figure 6A). Western blot confirmed the presence of exosome markers (TSG101, CD9, CD81), the absence of Calnexin, and the enrichment of TAGLN2 in *TAG2*‐exo (Figure 6B). Confocal microscopy confirmed efficient uptake of PKH26‐labeled exosomes by ECs (Figure 6C). To definitively establish that exosome‐encapsulated TAGLN2 is the active mediator, we performed Co‐IP using exosomes derived from BGC‐823 cells stably expressing 3×Flag‐TAGLN2 or EGFP (control). Following co‐culture of these exosomes with ECs for 18 h, cell lysates were subjected to IP using an anti‐Flag antibody. Notably, exosomal Flag‐TAGLN2, but not the control, specifically pulled down endogenous NRP1, SEMA4D, SP1, and c‐Jun in recipient ECs (Figure 6D), which provides direct biochemical evidence for the physical interaction of exosomal TAGLN2 with core components of its signaling axis in target cells.

Treatment of ECs with exosomes (5 µg/mL, 24 h) showed that *NC*‐exo, sh*NC*‐exo, and *TAG2*‐exo enhanced cell migration, tube formation (Figure 6E), and the expression of pro‐angiogenic factors (TAGLN2, NRP1, SEMA4D, VEGFR2, CD31, and vWF), with the most potent effects observed with *TAG2*‐exo (Figure 6F). In contrast, sh*TAG2*‐exo suppressed these processes. Mechanistically, *NC*‐exo, sh*NC*‐exo, or *TAG2*‐exo treatment significantly downregulated key junctional proteins (Occludin, ZO‐1, Claudin 5, VE‐cadherin, E‐cadherin), and upregulated N‐cadherin, with effects correlated to exosomal TAGLN2 content (Figure 6G), and disrupted Occludin localization at cell–cell contacts, as confirmed by immunofluorescence (Figure 6H). Furthermore, exosomal TAGLN2 inhibited Hippo signaling, decreased levels of MST1/2, *p*‐MST1/MST2, LATS1, *p*‐LATS1 and *p*‐YAP, while increased Ajuba, PTPN14, YAP, TAZ and β‐catenin (Figure 6I). sh*TAG2*‐exo elicited the opposite effect. Consistent results were observed using exosomes from other GC cell lines (MGC‐803, SGC‐7901), which similarly elevated TAGLN2, NRP1, SEMA4D, activated YAP, and disrupted endothelial junctions (Figure 6J).

We next assessed whether exosomal TAGLN2 similarly signals independently of PlexinB1. As expected, both *NC*‐exo and *TAG2*‐exo promoted YAP activation and junctional disassembly, with a markedly greater magnitude induced by *TAG2*‐exo in si*NC* cells. Critically, and in direct parallel to the genetic data in Figure 5I, *TAG2*‐exo potently suppressed *p*‐MYPT1/*p*‐MLC and *p*‐YAP while activating YAP/β‐catenin and suppressing Occludin/ZO‐1, and these effects remained strongly sustained even in PlexinB1‐deficient cells when compared to PBS or *NC*‐exo treatment (Figure 6K). These results directly demonstrate that exosome‐delivered TAGLN2 engages the identified ternary complex axis and operates independently of the canonical SEMA4D‐PlexinB1 pathway.

### 2.7 Exosomal TAGLN2 Promotes Tumor Vascular Dysfunction, Progression, and Metastasis In Vivo

**FIGURE 7:**
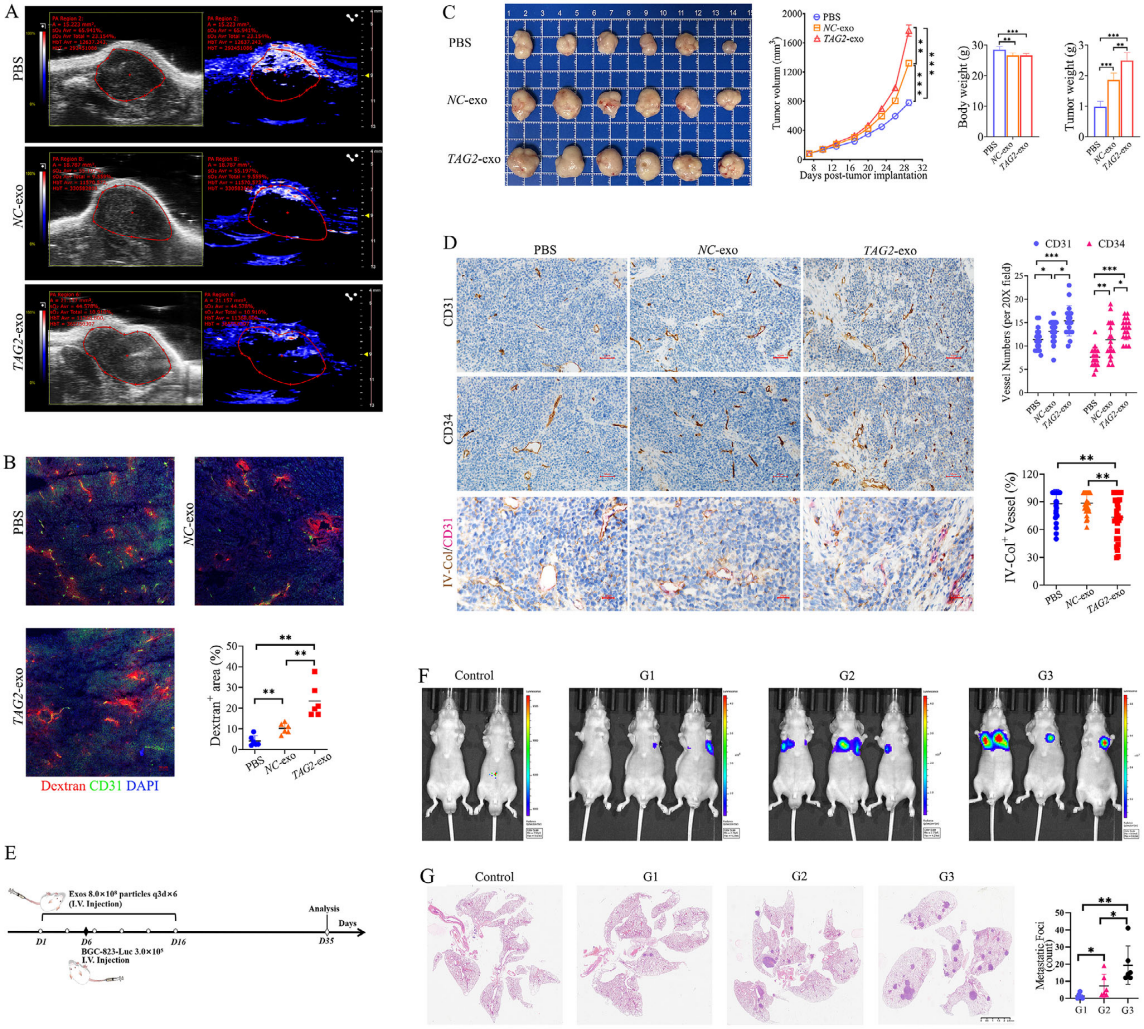
GC‐derived exosomal TAGLN2 promotes tumor vascular dysfunction, progression, and metastasis in vivo. (A) In vivo photoacoustic imaging of tumor vascular function in mice bearing BGC‐823 xenografts receiving intratumoral injections of PBS, *NC*‐exo, or *TAG2*‐exo. Average tumor oxygen saturation (sO2 Avr) and total hemoglobin content (HbT Avr) are shown. (B) Vascular permeability assessed by TRITC‐dextran extravasation. Representative fluorescence images and quantification of dextran leakage area related to CD31^+^ vessels. (C) Exosomal TAGLN2 promotes tumor growth. Tumor growth curves, final tumor weights, and corresponding mouse body weights are presented (*n* = 6). (D) Analysis of tumor vasculature. Representative IHC images and quantification of microvessel density (CD31^+^, CD34^+^) and vascular integrity by Collagen IV (IV‐Col)/CD31 dual staining. (E) Schematic of the experimental design for the BGC‐823‐Luc lung metastasis model. (F) Representative in vivo bioluminescence imaging of lung metastatic burden. Mice received tail vein injections of PBS (G1), *NC*‐exo (G2), or *TAG2*‐exo (G3) every three days for six doses (q3d×6). (G) Representative H&E‐stained lung sections and quantification of metastatic foci (*n* = 6). Data are presented as mean ± SD. Significance vs. PBS control: ns, not significant; ^*^ *p*< 0.05, ^**^ *p*< 0.01, ^***^ *p*< 0.001. Lines with symbols denote pairwise comparisons.

To investigate the in vivo function of exosomal TAGLN2, mice bearing subcutaneous BGC‐823 xenografts received intratumoral injections of *NC*‐exo or *TAG2*‐exo. Photoacoustic imaging (PAI) revealed a significant reduction in both average tumor oxygen saturation (sO_2_ Avr) and total hemoglobin content (HbT Avr) in the *TAG2*‐exo group, indicating impaired vascular perfusion and elevated hypoxia (Figure 7A). Consistently, TRITC‐dextran extravasation assays revealed severe endothelial barrier disruption in *TAG2*‐exo‐treated tumors, evident from widespread fluorescent dextran aggregation and a significantly larger dextran‐positive area (*p*< 0.01, Figure 7B).

Concurrently, exosome administration markedly promoted tumor growth, as reflected in tumor progression curves and final weight. Tumor weights in the *NC*‐exo and *TAG2*‐exo groups were approximately 1.9 and 2.5 fold greater, respectively, than in the PBS control group (Figure 7C). A concomitant decrease in body weight suggested systemic compromise due to tumor burden. Histologically, tumors from the *TAG2*‐exo group displayed increased microvessel density (CD31^+^ and CD34^+^ vessels), which were predominantly small‐diameter and immature. However, dual staining for Collagen IV (IV‐Col) and CD31 revealed a severe loss of vascular basement membrane integrity (Figure 7D). Collectively, these findings indicate that exosomal TAGLN2 induces aberrant, leaky vasculature, thereby exacerbating tumor hypoxia and progression.

To further assess the pro‐metastatic role of exosomal TAGLN2, we established a lung metastasis model via intravenous injection of BGC‐823‐Luc cells. IVIS imaging showed that both types of GC‐derived exosomes significantly enhanced the metastatic burden compared to the PBS control, with the *TAG2*‐exo group exhibiting the most intense bioluminescence, indicating markedly accelerated growth of lung metastases (Figure 7E,F). Correspondingly, H&E staining confirmed a substantially greater number of metastatic foci in the lungs of *TAG2*‐exo‐treated mice compared to the *NC*‐exo group (Figure 7G), underscoring the potent pro‐metastatic effect of exosomal TAGLN2.

### 2.8 Therapeutic Targeting and Circulating Exosomal TAGLN2 as a Biomarker in Gastric Cancer

**FIGURE 8:**
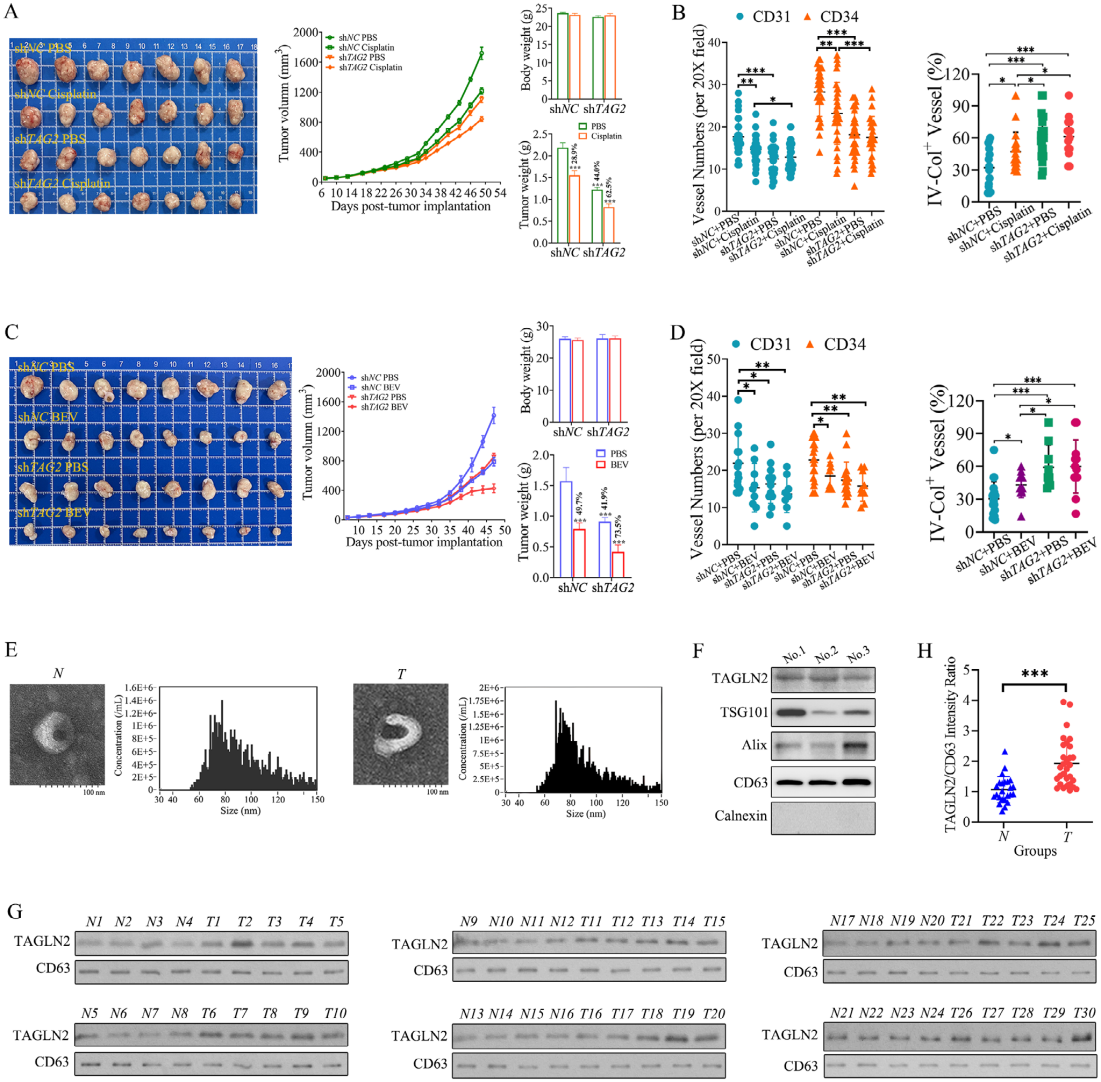
Therapeutic targeting and circulating exosomal TAGLN2 as a biomarker in gastric cancer. (A∼D) Therapeutic efficacy of targeting the TAGLN2 axis combined with cisplatin or bevacizumab. Mice bearing tumors from BGC‐823 cells (sh*NC* or sh*TAG2*) received intraperitoneal cisplatin (3 mg/kg q4d, *n* = 7), bevacizumab (BEV; 2 mg/kg twice weekly, *n* = 8), or PBS. Representative tumor images, growth curves, final body weight (without tumor), tumor weights, and quantification of CD31^+^, CD34^+^, and IV‐Col/CD31 dual staining are shown for cisplatin‐ (A, B) and BEV‐treated groups (C, D), respectively. (E) Representative TEM images and NTA showing the characterization of exosomes isolated from non‐cancer control (*N*) and GC patient (*T*) sera. (F) Western blot analysis of exosomal markers and TAGLN2 in randomly selected serum samples. (G) Immunoblots of TAGLN2 and the loading control CD63 from all individual samples (*n* = 24 non‐cancer controls; *n* = 30 GC patients). (H) Semi‐quantitative analysis of the TAGLN2/CD63 ratio, demonstrating a significant elevation in GC patient serum. Data are presented as mean ± SD. Significance vs. respective control: ns, not significant; ^*^ *p*< 0.05, ^**^ *p*< 0.01, ^***^ *p*< 0.001. Lines with symbols denote pairwise comparisons.

To evaluate the therapeutic potential of targeting the TAGLN2 axis, we combined *TAGLN2* knockdown in BGC‐823 cells with conventional chemotherapeutic and targeted agents (cisplatin or bevacizumab/BEV) in subcutaneous xenograft models. As shown in Figure 8A, *TAGLN2* knockdown alone achieved a 44 % tumor growth inhibition (TGI). Cisplatin monotherapy resulted in a 28.9 % TGI, while the combination of *TAGLN2* knockdown with cisplatin yielded a synergistic effect, significantly enhancing the efficacy to 62.5 % TGI. Consistent with its role in vascular regulation, *TAGLN2* knockdown markedly reduced the density of CD31^+^ and CD34^+^ microvessels and increased the coverage of the vascular basement membrane, as evidenced by a significantly elevated IV‐Col/CD31 percentage, indicating improved vascular maturation and stability (Figure 8B).

Similarly, in the anti‐angiogenic therapy model (Figure 8C), both BEV monotherapy and *TAGLN2* knockdown alone attenuated tumor growth compared to the control group, achieving TGI rates of 49.7 % and 41.9 %, respectively, with no significant difference between the two single treatments. The most profound suppression was observed in the combination therapy group (sh*TAG2*+BEV), which displayed the slowest growth kinetics, entered a plateau phase after 40 d, and achieved a final TGI of 73.5 %. IHC analysis confirmed the cooperative effect on vascular normalization. Both similarly reduced the density of CD31^+^ and CD34^+^ microvessels. Notably, while BEV monotherapy significantly increased vascular basement membrane coverage (IV‐Col/CD31 %) compared to the PBS control, TAGLN2 knockdown induced a substantially greater increase in coverage than BEV alone. The combination treatment did not further enhance coverage beyond the level achieved by TAGLN2 knockdown alone (Figure 8D). This indicates that TAGLN2 knockdown is particularly effective in promoting vascular maturation by enhancing basement membrane integrity, a key aspect of vascular normalization.

To validate the clinical relevance of our findings, we detected exosomal TAGLN2 in the serum of GC patients. Exosomes were efficiently isolated from 1 mL of serum using a Membrane Sensing Peptide Magnetic Bead Separation System. TEM and NTA confirmed the isolation of typical cup‐shaped vesicles with homogenous size distributions (average diameters: 88.3 nm for non‐cancer controls vs. 92.4 nm for GC patients) at comparable particle concentrations (9.76 × 10^7^ particles/mL and 9.85 × 10^7^ particles/mL, respectively, Figure 8E). Western blot analysis of randomly selected samples validated the specific presence of exosomal markers (CD63, TSG101, Alix) and TAGLN2, with the absence of the negative marker Calnexin (Figure 8F).

Purified exosomes from a cohort of 24 non‐cancer controls and 30 GC patients (clinical characteristics summarized in Table S2) were lysed and analyzed under strictly standardized conditions. All individual immunoblots are presented. For semi‐quantification, the band intensity of TAGLN2 was normalized to that of CD63 within each sample, and the TAGLN2/CD63 ratio was calculated (Figure 8G,H). Quantitative analysis revealed a highly significant elevation of exosomal TAGLN2 in GC patient serum compared to controls (*p*< 0.001). This direct clinical evidence confirms that exosomal TAGLN2 is not only a promising non‐invasive biomarker but also a key driver of the vascular dysfunction and metastasis previously characterized in our cellular and animal models.

## 3 Discussion

This study elucidates a novel signaling axis through which GC‐derived exosomal TAGLN2 reprograms ECs to fuel metastasis. We demonstrate that exosomal TAGLN2 nucleates a cytoplasmic NRP1/SEMA4D ternary complex, which potently activates YAP via a PlexinB1‐ and RhoA/ROCK‐independent mechanism. This axis drives a coherent pro‐metastatic program in endothelium, characterized by EndoMT, junctional disintegration, hyperpermeability, and angiogenesis. The clinical and therapeutic relevance of this pathway is underscored by the elevation of TAGLN2 in circulating exosomes from GC patients and the significant suppression of tumor progression achieved by targeting this axis.

TAGLN2 is a 22‐kDa actin stress fiber‐associated protein that stabilizes actin filaments. Although TAGLN2 overexpression has been reported in multiple cancers, its functional role, especially in tumor microenvironment communication, remains poorly understood. Consistent with its vesicle‐associated localization predicted by GO analysis, TAGLN2 has been detected as an exosomal cargo in proteomic studies [18, 19], but a systematic understanding of its exosome‐mediated biological functions is lacking. Here, we demonstrate that TAGLN2 is not only upregulated in GC cells and TECs but is also selectively packaged into tumor‐derived exosomes for delivery to recipient ECs. Indeed, TAGLN2 was detected in exosomes derived from BGC‐823, MGC‐803, and SGC‐7901 cells, and its abundance correlated with TAGLN2‐overexpression or knockdown. Its delivery to ECs is both necessary and sufficient to induce the key vascular abnormalities that facilitate metastasis: angiogenesis, EndoMT, and Barrier breakdown. This positions exosomal TAGLN2 not merely as a biomarker, but as an active master regulator of a pro‐metastatic vascular switch, providing a direct mechanistic link between tumor‐derived vesicles and the promotion of metastatic spread.

The vascular receptor Neuropilin 1 (NRP1) emerged as the critical signaling nexus. While NRP1 is a well‐characterized transmembrane glycoprotein with pleiotropic roles in nervous system development, T cell memory checkpoint, angiogenesis, and vascular permeability [24, 25], its regulation and context‐specific functions remain incompletely understood. NRP1 expression is modulated by transcription factors such as AP1, SP1, C/EBPβ, AP2, AP4, STAT, and HIF‐1α [20, 26], and it regulates vascular integrity through mechanisms like controlling the endocytic turnover of active α5β1 integrins and VE‐cadherin [27]. Here, we unveil a novel, dual‐tiered regulatory mechanism orchestrated by TAGLN2. Specifically, TAGLN2 not only transcriptionally upregulates NRP1 by enhancing the recruitment of the SP1/c‐Jun to its promoter, but also physically stabilizes NRP1 protein in the cytoplasm, thereby facilitating its assembly into a functional complex with SEAM4D. This coordinated action ensures both ample production and prolonged activity of NRP1, representing a sophisticated strategy to lock the pathway into a sustained activated state.

The downstream effector of this axis is YAP, a pivotal regulator of angiogenesis, EndoMT, and junctional dynamics. Previous studies have shown that VEGF‐A/NRP1 signaling promotes YAP accumulation [28], whereas mechanical transduction through NRP1 suppresses YAP expression via LATS1 and promotes its nuclear translocation by attenuating the NRP1‐YAP interaction [22]. Furthermore, hyperactive YAP/TAZ signaling is known to inhibit tight junction assembly in vascular ECs [29, 30]. Our study fundamentally redefines NRP1 from a membrane co‐receptor to a cytoplasmic hub within a specific macromolecular assembly. We demonstrate that the formation of the TAGLN2/NRP1/SEMA4D ternary complex reprograms NRP1's function, orchestrating two coordinated actions that converge on potent YAP activation. First, the complex sequesters NRP1, displacing it from its interaction with YAP and thereby relieving YAP from cytoplasmic retention. Second, and more broadly, the formation of this complex leads to a comprehensive suppression of the Hippo signaling pathway, as evidenced by the coordinated downregulation of its core kinases and phosphorylated effectors. Consistent activation of this axis resulted in marked downregulation of multiple Hippo pathway components, including MST1/2, *p*‐MST1/MST2, LATS1, *p*‐LATS1, and *p*‐YAP. This suppression created a permissive environment for YAP/TAZ activation, further amplified by upstream positive regulators such as Ajuba and F‐actin polymerization. Consequently, the protein levels of YAP, its paralog TAZ, and the downstream target β‐catenin were significantly elevated. The essential role of NRP1 in this regulatory switch was confirmed, as its knockdown restored Hippo pathway activity in *TAGLN2*‐overexpressing ECs. Conversely, pharmacological inhibition of the pathway downstream at LATS1/2 with GA‐017 effectively reversed the axis‐induced loss of key junction proteins. Collectively, our results establish that the TAGLN2/NRP1/SEMA4D axis drives vascular permeability and angiogenesis by activating YAP.

SEMA4D is overexpressed in various human malignancies. Typically, as a membrane‐bound ligand, it signals through its high‐affinity receptor PlexinB1 to activate the RhoA/ROCK pathway, thereby driving EMT, angiogenesis, and metastasis [31]. It facilitates the internalization of adhesion proteins such as VE‐cadherin and N‐cadherin and induces pericyte loss, thereby exacerbating vascular permeability [23]. It can also be cleaved into a soluble form (sSEMA4D), exerting multifaceted effects on different cell types. Consequently, therapeutic strategies targeting the SEMA4D/PlexinB1 axis, such as the monoclonal antibody VX15/2503, have shown promise [32]. However, our study reveals that SEMA4D can be co‐opted into a fundamentally different signaling paradigm with two distinct structural roles. First, within the cytoplasm, SEMA4D acts as a critical molecular bridge, mediating the physical interaction between TAGLN2 and NRP1 to ensure the assembly and stability of the ternary complex. Second, SEMA4D competitively binds to NRP1, displacing YAP from its cytoplasmic tether and thereby facilitating YAP release. This reconfigured complex completely uncouples SEMA4D from its canonical pathway. We demonstrate that this axis (1) functions without generating sSEMA4D, (2) remains fully active upon PlexinB1 genetic ablation, and (3) operates independently of and even suppresses the RhoA/ROCK pathway. Thus, we have identified a non‐canonical signaling module in which SEMA4D ensures complex assembly and initiates YAP release, while NRP1 is repurposed as a cytoplasmic hub to orchestrate dual YAP activation, collectively driving vascular dysfunction.

Tumor‐derived small EVs serve as critical biomarkers, facilitating early detection, diagnosis, therapeutic intervention, and prognostic evaluation of metastatic risk [33]. Our study profoundly extends this paradigm by establishing that exosomal TAGLN2 is not merely a passive indicator but a central functional driver of metastasis. We demonstrated that exosomal TAGLN2 significantly promotes tumor growth and lung metastasis in vivo, which was accompanied by a pathological angiogenic switch. This switch was characterized by an increase in immature microvessel density concurrent with a severe loss of vascular basement membrane integrity. The resulting structural defects led to reduced intratumoral oxygenation and elevated vascular permeability, thereby accelerating tumor progression.

Therapeutically, targeting the TAGLN2 axis offers a synergistic strategy. This approach simultaneously addresses both the intrinsic oncogenicity of tumor cells and the abnormal tumor vasculature. Specifically, it directly inhibits tumor cell proliferation, while also reprogramming the tumor vasculature toward normalization. Our assessment of the vascular remodeling induced by TAGLN2 targeting, which encompasses both the reduction of aberrant microvessel density and the enhancement of basement membrane coverage, reveals a distinct advantage over conventional anti‐angiogenic therapy. Although both BEV and TAGLN2 knockdown reduced microvessel density, TAGLN2 inhibition uniquely and potently enhances vascular coverage. The combination therapy achieves no further improvement beyond the effect of TAGLN2 knockdown alone. This dual efficacy, which combines direct tumor cell killing with superior vascular normalization, not only validates the blockade of exosomal TAGLN2 delivery as a central pathogenic mechanism but also provides a comprehensive therapeutic rationale.

Concurrently, we provide foundational clinical evidence that positions this functional axis for translational application. We show, for the first time, that the protein level of exosomal TAGLN2 is specifically and significantly elevated in the serum of GC patients, establishing it as a promising non‐invasive biomarker. This finding transforms the perception of exosomal TAGLN2 from a correlative signal to an active pathogenic vehicle. Through this vehicle, tumor cells systemically deliver a pro‐metastatic instruction to remodel the vascular microenvironment for distant dissemination. Building upon this, a critical future direction is the prospective, multi‐center validation of circulating exosomal TAGLN2 levels against key clinical endpoints, including metastatic risk, overall survival, and recurrence. The implementation of more sensitive, high‐throughput quantitative platforms, such as digital ELISA or single‐vesicle analysis, will be crucial for robust measurement in these expanded clinical settings.

In summary, our study delineates a complete and actionable signaling axis from tumor exosomes to vascular reprogramming. We demonstrate that GC‐derived exosomal TAGLN2 nucleates a cytoplasmic TAGLN2/NRP1/SEMA4D ternary complex, which then activates YAP through a novel mechanism independent of the canonical PlexinB1‐RhoA/ROCK pathway. This activation is achieved via a dual mechanism: competitively liberating YAP from NRP1 sequestration while simultaneously suppressing its Hippo pathway‐mediated degradation. Clinically, this axis holds dual relevance: exosomal TAGLN2 serves as an elevated and functionally relevant biomarker in GC patient serum, and its targeting synergizes with therapies not only to suppress tumor growth but also to inhibit pathological angiogenesis and promote vascular normalization. Collectively, our work defines a novel exosome‐to‐YAP signaling bridge, positioning the TAGLN2/NRP1/SEMA4D/YAP module as an integrated diagnostic and therapeutic target with transformative potential for metastatic GC.

## 4 Materials and Methods

### 4.1 Cell Lines and Animal Model

The following cell lines were used in this study: MKN45 (RRID:CVCL_0434), BGC‐823 (RRID:CVCL_3360), SGC‐7901 (RRID:CVCL_0520), MGC‐803 (RRID:CVCL_5334), HMEC‐1 (RRID:CVCL_0307), and EA.hy926 (RRID:CVCL_3901). All cell lines were authenticated and confirmed mycoplasma‐free prior to experimental use. Cells were maintained in RPMI 1640 medium supplemented with 10 % fetal bovine serum (FBS, Sigma, USA), 100 U/mL penicillin, and 100 µg/mL streptomycin, except for HMEC‐1 and EA.hy926, which were maintained in DMEM and DMEM/F12 medium, respectively. Transient transfection of plasmids and siRNAs was carried out using Lipofectamine 2000 or Lipofectamine RNAi MAX (Thermo Fisher Scientific, USA), respectively. For stable gene expression, cells were transduced with a lentiviral expression vector (pGLV2‐U6‐Puro) and selected with puromycin. Primers sequences for plasmid construction and siRNA target sequences are listed in Tables S3 and S4.

Animal care and handling were performed in compliance with the Guidelines for the Care and Use of Laboratory Animals, and the animal study protocol was approved by the Institutional Animal Care and Use Committee of Xiamen University (Approval No. XMULAC20220268). Systemic *Tagln2* knockout mouse models (*Tagln2* ^−/−^) were generated using the CRISPR/Cas9 system by the Model Animal Research Center of Xiamen University Medical College. BALB/c nude mice were maintained and treated under specific pathogen‐free conditions in accordance with the aforementioned guidelines.

### 4.2 Tissue Microarray (TMA) and Multiplex Immunofluorescence

TMA of GC (Cat#HStmA180Su11), comprising 90 cases of tumor tissues with paired adjacent normal tissues, was commercially obtained from Shanghai Outdo Biotech Co., Ltd (Shanghai, China). The cohort includes comprehensive clinicopathological characterization and survival data. The provider is authorized by the Human Genetic Resource Administration of China (HGRAC) (Approval No. [2022] BC0020). The development and application of this specific TMA product were approved by the provider's Ethics Committee (Approval No. SHYJS‐CP‐1710001). Multiplex immunofluorescence was carried out using an Opal 7‐color Manual IHC Kit (NEL801001KT, PerkinElmer, USA) and VECTASHIELD HardSet Antifade Mounting Medium (H‐1400, Vector Labs). Methods were carried out based on our previously established protocols [17]. Briefly, TMA sections were blocked and incubated with one of the following antibodies for 1 h: anti‐TAGLN2 (1:500, Proteintech, China), anti‐CD34 (1:100, DAKO, Denmark), and anti‐CK (1:2, Abcarta Medtech Co., Ltd., China). The multiplex antibody panel was optimized as follows: TAGLN2/Opal 520, CK/Opal 570, CD34/Opal 690. Finally, slides were counterstained with DAPI and coverslipped using antifade mounting medium. Panoramic multispectral scanning and quantitation analyses of TMA were performed using the TissueFAXS Spectra Systems and StrataQuest analysis software (TissueGnostics).

### 4.3 Exosomes Isolation and Characterization

GC cell lines were cultured in RPMI‐1640 medium. After 48 h, the culture medium was sequentially centrifuged at 300 × *g* for 10 min, 2000 × *g* for 30 min, and 10 000 × *g* for 45 min at 4°C. The resulting supernatant was then filtered through a 0.45 µm filter and ultracentrifuged at 100 000 × *g* for 70 min at 4°C (Hitachi CP100MX ultracentrifuge, Japan). The pellet was resuspended in PBS and again ultracentrifuged under the same conditions. The final exosome pellet was collected and quantified using a BCA Protein Assay kit (Millipore, USA). Exosomes' identity and purity were validated by transmission electron microscopy (Hitachi HT‐700, Japan), Nano‐flow cytometry (NanoFCM N30E, China), and western blot for positive markers (TSG101, CD9, CD63, Alix, and CD81) and the negative marker Calnexin. For uptake experiments, exosomes were labeled with PKH26 (Sigma, USA) according to the manufacturer's instructions and incubated with EA.hy926 cells for 12 h. Internalization was visualized using a Zeiss LSM780 confocal microscope (Carl Zeiss GmbH, Germany) with a 63× objective.

### 4.4 Function Assays for the Tumor‐Derived Exosomal TAGLN2

ECs were incubated with exosomes (5 µg/mL) derived from BGC‐823 (with stable *TAGLN2* knockdown or overexpression: sh*NC*‐exo*/*sh*TAG2*‐exo, *NC*‐exo/*TAG2*‐exo), MGC‐803, or SGC‐7901 cells for 24 h. The effects of exosomal TAGLN2 on EC migration and tube formation were assessed by using the migration assay, the tube formation assay, and the in vivo Matrigel plug assay.

For endothelial monolayer permeability assay, ECs were grown to confluence on 0.4 µm polyethylene terephthalate transwell filters (BD Biosciences, NJ) in 24‐well plates. Rhodamine‐dextran (average MW ≈ 70 000, Sigma) was applied to the upper chamber at a final concentration of 20 mg/mL. The fluorescence intensity in the lower chamber was measured at timed intervals by collecting 40 µL aliquots and detecting with a microplate reader (Infinite M1000, Tecan, Switzerland) at 544/590 nm excitation/emission wavelengths.

For transendothelial migration assays, EC monolayers were established in the upper chamber of transwell inserts (8 µm pore size) in 24‐well plates. CM‐DiI‐labeled or GFP‐expressing GC cells were added, and after 24 h, the number of migrated cells in the lower chamber was quantified under a fluorescence microscope.

For tumor‐endothelium adhesion assays, 1 × 10^5^ GFP‐expressing GC cells were co‐cultured with EC monolayers in 12‐well plates for 20 min at 37°C. Non‐adherent cells were removed by gentle washing three times with PBS, and adherent fluorescent cells were counted under a microscope.

### 4.5 Western Blot and ELISA Analysis

Cells and exosomes were lysed using RIPA lysis buffer supplemented with protease and phosphatase inhibitors. Proteins were separated by SDS‐PAGE and transferred to PVDF membranes. After locking, the membranes were incubated overnight at 4°C with specific primary antibodies. Protein bands were visualized using enhanced chemiluminescence reagents (Bio‐Rad). Detailed information on primary antibodies used is provided in Table S5. The concentration of soluble SEMA4D (sSEMA4D) in cell culture supernatant was quantified using a human Semaphorin 4D ELISA Kit (Invitrogen, Cat#EH405RB) following the manufacturer's protocol.

### 4.6 Co‐IP, ChIP, and GST‐Pull Down Assays

Co‐IP assays were performed using a Pierce Co‐IP kit (Thermo Fisher Scientific, Cat#26149). Antibodies against TAGLN2, c‐Jun, SP1, NRP1, YAP, or Flag were immobilized onto the coupling resin. After incubation with cell lysates, the resin was washed, and bound proteins were eluted. Both co‐immunoprecipitates and input lysates were detected by Western blot.

ChIP was conducted using a Pierce ChIP assay kit (Thermo Fisher Scientific). Precipitated DNA was analyzed by qRT‐PCR and agarose gel electrophoresis. Primer pairs spanning potential TAGLN2‐binding regions in the human *NRP1* promoter were listed in Table S3.

For GST pull‐down assays, plasmids encoding GST‐TAGLN2, GST‐GFP, His‐NRP1, HA‐SEMA4D, GST‐SEMA4D, or Flag‐TAGLN2 were transfected into *E. coli* for fusion protein expression. Protein interaction studies were conducted using the Pierce GST Protein Interaction Pull‐Down Kit (Thermo Fisher Scientific, Cat#21516). Briefly, the GST‐tagged bait protein was immobilized on equilibrated glutathione agarose resin at 4°C for 2 h with gentle agitation. After centrifugation and five washes, the resin was incubated with prey protein samples at 4°C for 2 h, followed by repeated washing. Bound proteins were eluted with glutathione elution buffer and detected by Western blot.

### 4.7 Dual Luciferase Reporter Assay

To assess *NRP1* promoter activity, EA.hy926 cells were transiently co‐transfected with a luciferase reporter plasmid containing the *NRP1* promoter region (−823/+79) along with pcDNA3.1‐*C/EBPβ*, pcDNA3.1‐*SP1*, or pcDNA3.1*‐c‐Jun*, respectively. Parallel experiments were performed in cells subjected to knockdown of *TAGLN2*, *c‐Jun*, or *SP1*. The empty pGL3‐basic vector was used as a negative control. Luciferase activity was measured using the Dual‐Luciferase Reporter Assay System (Promega) according to the manufacturer's instructions.

### 4.8 Immunohistochemical (IHC) Staining

IHC staining was performed using the EnVision + System‐HRP kit. Analysis/scoring of IHC data was performed by two certified pathologists; the final score represents the average of their assessments. Antibodies used included: anti‐CD31 (Abcam, Cat#ab28364), CD34 (Abcam, Cat#ab8158), Occludin (Proteintech, Cat#13409‐1‐AP), VE‐cadherin (BD, Cat#555289), N‐cadherin (R&D, Cat#AF6426), and Collagen IV (Arigobio, Cat#ARG21968). The percentage of positive (PP) cells was scored as 0 (PP≤ 5 %), 1 (6 %≤ PP≤ 25 %), 2 (26 %≤ PP≤ 50 %), 3 (51 %≤ PP≤ 75 %), and 4 (PP ≥76 %). The intensity of staining (IS) was scored as: 0 (negative), 1 (weak, light yellow), 2 (moderate, brown yellow), 3 (strong, brown). The immunoreactive score (IRS) was calculated as IRS = PP × IS.

### 4.9 In Vivo Evaluation of Tumor Promotion

For subcutaneous xenograft studies, BALB/c nude mice (male, 5‐week‐old) were injected subcutaneously with 2 × 10^6^ BGC‐823 cells suspended in 0.2 mL PBS. Tumor volume and body weight were monitored every three days. Tumor volume was calculated using the following equation:

(1) $$V=d_{1}\times {\left(d_{2}\right)}^{2}\times 0.5$$

where d_1_ represents the longest diameter and d_2_ the perpendicular diameter. Once tumors were established, mice were randomly divided into three groups (Day 7) and received intratumoral injections of PBS, *NC*‐exo, or *TAG2*‐exo (7 × 10^8^ particles per injection) every three days for a total of four injections. At the endpoint, tumors were excised, weighed, and snap‐frozen in liquid nitrogen for further histological analysis.

To assess vascular permeability in vivo, mice bearing subcutaneous tumors were intravenously injected with TRITC‐Dextran (70 kDa, 100 mg/kg; Sigma T1162) at day 21. At 3 h post‐injection, mice were perfused, and tumors were harvested for frozen sectioning and immunofluorescence analysis.

PAI co‐registered with ultrasound (US) was performed using a laser‐integrated high‐frequency US system (Vevo LAZR‐X, FujiFilm VisualSonics Inc., Toronto, Canada). 3D B‐mode US images were first acquired to determine tumor volume measurements. Subsequently, PAI datasets were obtained for quantitative mapping of whole tumor oxygen saturation (sO_2_) and total hemoglobin concentration (Hbt) using the following parameters: frequency: 21 MHz, depth: 22.00 mm, width: 21.04 mm, wavelength: 750/850 nm, and acquisition mode: sO_2_/HbT.

For the experimental lung metastasis model, BALB/c nude mice (5‐week‐old) received tail vein injection of PBS, *NC*‐exo, or *TAG2*‐exo (*n* = 6, 8×10^8^ particles per injection) every three days, for a total of six injections. One day prior to the third exosome injection, all mice were inoculated via the tail vein with 3 × 10^5^ BGC‐823‐Luc cells. Metastatic progression was monitored using an in vivo luciferase‐based IVIS imaging system. At the experimental endpoint, lungs were harvested, and metastatic nodules were quantified. Lung sections were stained with Hematoxylin and Eosin (H&E) for histological evaluation.

### 4.10 In Vivo Therapeutic Studies in Subcutaneous Xenograft Models

To evaluate therapeutic efficacy, a subcutaneous xenograft model was established by inoculating mice with 2×10^6^ BGC‐823 cells (with or without *TAGLN2* knockdown) suspended in 0.2 mL PBS. When tumor volumes reached 60–100 mm^3^, mice were randomly assigned to receive the following intraperitoneal treatments: cisplatin (Jiangsu Haosen Pharmaceutical Group, China; 3 mg/kg every 4 days), bevacizumab (Chiatai Tianqing Pharmaceutical Group, China; 2 mg/kg twice weekly), or an equivalent volume of PBS as vehicle control. After the treatment period, mice were euthanized. Tumors were excised, weighed, and immediately snap‐frozen in liquid nitrogen for subsequent histological analysis.

### 4.11 Clinical Serum Exosome Isolation and Detection of Exosomal TAGLN2

Serum samples were obtained from treatment‐naïve GC patients and non‐cancer controls at Zhongshan Hospital, Xiamen University, with written informed consent and ethics approval (Approval No. xmzsyyky2025‐172). Peripheral blood was collected in pro‐coagulation tubes, clotted for 30 min at room temperature, and centrifuged at 2000 × *g* for 15 min at 4°C. The serum supernatant was aliquoted and stored at −80°C until use.

For exosome isolation, frozen serum aliquots were thawed at 37°C and clarified by centrifugation at 15 000 × *g* for 20 min at 4°C. Exosomes were then purified from 1 mL of pre‐cleared serum using EXO‐Smart Membrane Sensing Peptide Magnetic Bead Exosome Separation System with the EXO‐Smart‐16 Isolation kit (Xiamen Lifeint Technology Co., Ltd., China), processing 16 samples per run. Briefly, serum was mixed with magnetic beads at a 20:1 (V:V) ratio, and subsequent binding, washing, and elution steps were performed automatically.

Purified exosomes were immediately lysed on ice for 30 min using 5 × RIPA‐type lysis buffer, supplemented with protease/phosphatase inhibitor cocktail and 1 mM PMSF, with intermittent vortexing every 10 min. The lysate was centrifuged at 12 000 × *g* for 10 min at 4°C, and the resulting supernatant was mixed with 5 × SDS‐PAGE loading buffer and denatured at 100°C for 10 min. To enhance detection sensitivity for low‐abundance exosomal proteins, membranes were incubated with primary antibodies against CD63, TSG101, Alix, Calnexin, and TAGLN2 for 48 h at 4°C, followed by incubation with HRP‐conjugated secondary antibodies. Signals were developed using an enhanced ECL substrate and captured by exposure to X‐ray film.

### 4.12 Statistical Analysis

Statistical analysis was performed using SPSS 20.0 software (SPSS Inc., USA). Clinical characteristics were compared using the Chi‐square test. The linear relationship between variables was assessed using Pearson's correlation coefficient. Quantitative data from experimental replicates are presented as the mean ± standard deviation (SD). Comparisons between two groups were performed using a two‐tailed, unpaired Student's *t*‐test or the Mann–Whitney *U*‐test. Paired samples were analyzed using a paired *t*‐test. A *p*‐value <0.05 was considered statistically significant.

## Author Contributions

Shuqi Yu, Jiajia Zhuo, Xiaoquan Hong, and Shihao Rao contributed equally to this work. Huiqin Zhuo and Huifang Peng conceived and designed the research. Shuqi Yu, Jiajia Zhuo, Shihao Rao, Xiaoquan Hong, Huifang Peng, and Huiqin Zhuo performed the experiments. Shuqi Yu and Xiaoquan Hong analyzed/scored the IHC results. Xiaoquan Hong, Yafang Ye, Dandan Kang, and Huifang Peng analyzed and interpreted the data. Huiqin Zhuo wrote and reviewed the manuscript. Huifang Peng and Huiqin Zhuo provided administrative, technical, or material support. All authors contributed to the article and approved the final manuscript.

## Ethics Approval

Animal protocols were reviewed and approved by the Institutional Animal Care and Use Committee of Xiamen University (Approval No. XMULAC20220268). The collection and use of human GC and non‐cancer serum samples were reviewed and approved by the Medical Ethics Committee of Zhongshan Hospital, Xiamen University (Approval No. xmzsyyky2025‐172).

## Conflicts of Interest

The authors declare no conflicts of interest.

## Supporting information




**Supporting File**: advs74800‐sup‐0001‐SuppMat.docx.

## Data Availability

The data that support the findings of this study are available in the supplementary material of this article.
