# Supplementary material for: Tumor‐Derived Exosomal TAGLN2 Promotes Metastasis by Inducing Vascular Permeability and Angiogenesis via the NRP1/SEMA4D/YAP Axis
Source: Adv Sci (Weinh). 2026 Mar 13;13(29):e21962. doi: 10.1002/advs.202521962 (PMC13205752; doi:10.1002/advs.202521962)
Supplement: Supplementary file 1 — Supporting File: advs74800‐sup‐0001‐SuppMat.docx. [file ADVS-13-e21962-s001.docx]

Figure S1


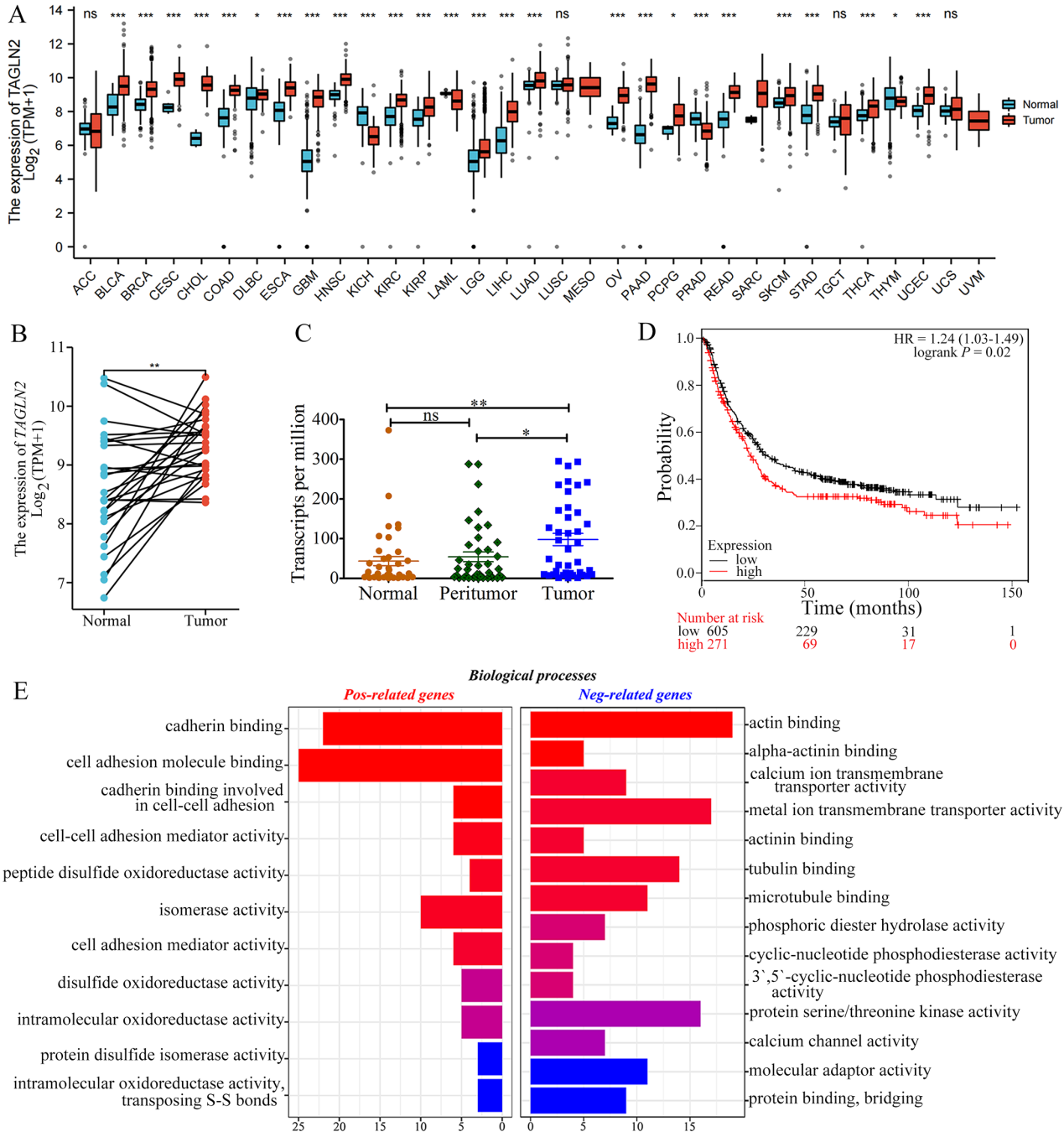


**Figure S1. Pan-cancer expression and clinical significance of TAGLN2 in gastric cancer**. (A) TAGLN2 mRNA expression across multiple human cancers from TCGA. (B, C) TAGLN2 mRNA expression in paired GC and adjacent normal tissues from the TCGA-STAD dataset (27 pairs) and our institutional gastrointestinal cancer biobank (40 pairs). (D) Survival analysis correlating high TAGLN2 expression with reduced overall survival in GC patients. (E) Functional enrichment analysis of TAGLN2 co-expressed and negatively correlated genes. ns, not significant; **P* < 0.05, ***P* < 0.01, ****P* < 0.001.

Figure S2


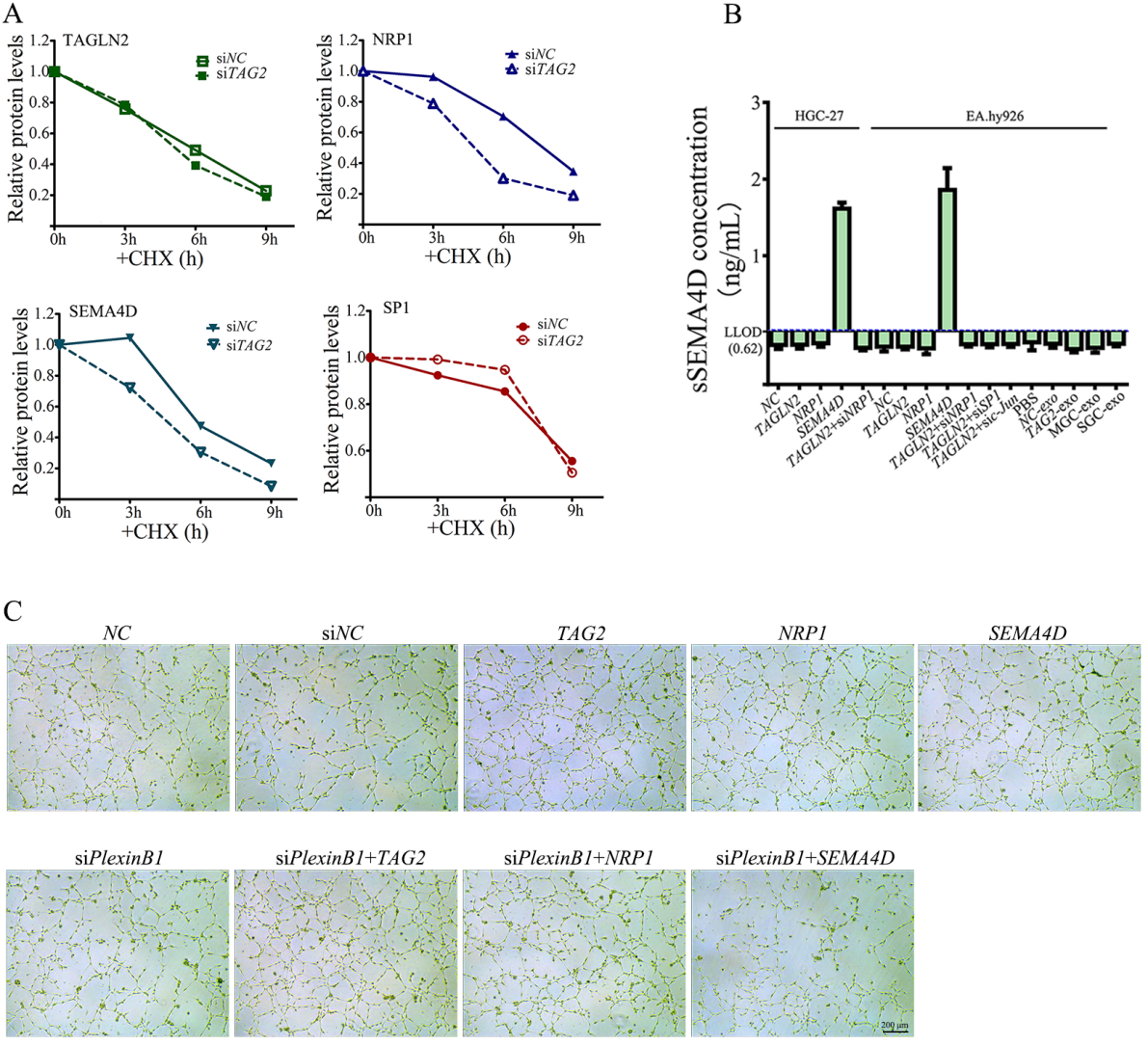


**Figure S2. TAGLN2 stabilizes the NRP1-SEMA4D complex and validates non-canonical pathway mechanisms.** (A) Quantification of protein stability by CHX chase assay. (B) Analysis of soluble SEMA4D (sSEMA4D) secretion by ELISA. Conditioned media were collected from HGC-27 or EA.hy926 cells subjected to the indicated genetic manipulations, or from ECs treated with specified GC-derived exosomes (*NC*-exo, *TAG2*-exo, MGC-exo, SGC-exo). The lower limit of detection (LLOD) was 0.62 ng/mL. (C) Representative images of capillary tube formation assays. EA.hy926 cells overexpressing *TAGLN2*, *NRP1*, or *SEMA4D* were assessed for tube-forming capacity with or without PlexinB1 knockdown (si*PlexinB1*). Data are presented as mean ± SD.

Figure S3


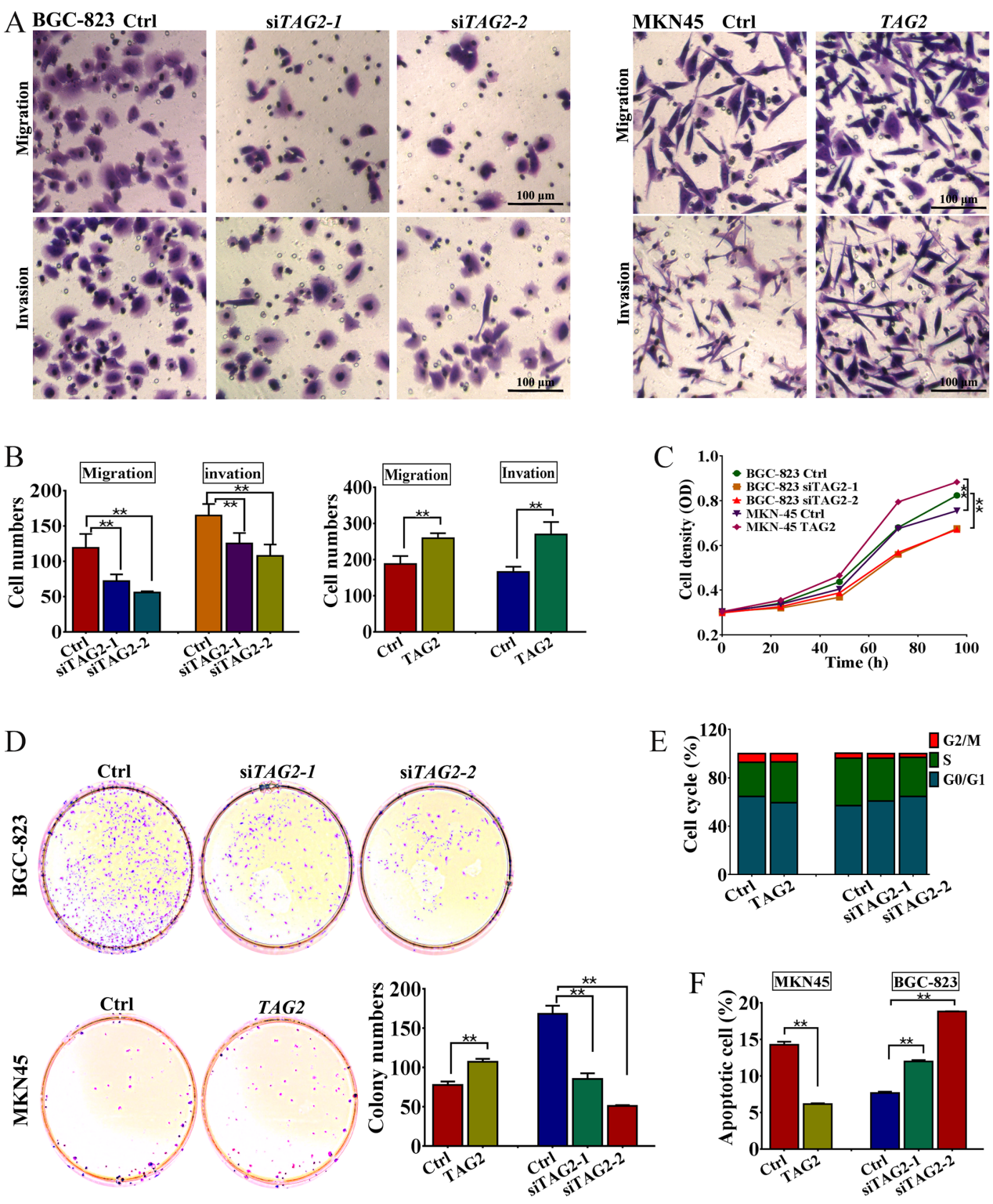


**Figure S3. Functional characterization of TAGLN2 in gastric cancer cell lines**. (A, B) Cell migration and invasion abilities assessed by Transwell assays in BGC-823 and MKN45 cells with *TAGLN2* knockdown or overexpression. (C) Cell proliferation measured by CCK-8 assay. (D) Clonogenic capacity evaluated by colony formation assay. (E) Cell cycle distribution analyzed by flow cytometry. (F) Apoptosis rates detected by flow cytometry following Annexin V/PI staining. Data are presented as mean ± SD. **P* < 0.05, ***P* < 0.01.

**Table S1 Correlation between intensity of TAGLN2 in TAGLN2^+^ CD34^+^ cells and clinical features**

| Features | Cases | Intensity of TAGLN2 in  TAGLN2^+^ CD34^+^ cells | |
| --- | --- | --- | --- |
|  |  | Pearson's R | *P* value |
| **Gender** | | | |
| Male | 61 | -0.04 | 0.708 |
| Female | 29 |  |  |
| **Age (years)** | | | |
| ≤ Average | 41 | 0.026 | 0.810 |
| > Average | 49 |  |  |
| **Stage** | | | |
| I-II | 2 | 0.033 | 0.760 |
| II | 14 |  |  |
| II-III | 18 |  |  |
| III | 42 |  |  |
| III-IV | 14 |  |  |
| **Tumor Size (cm)** | | | |
| ≤ 3 | 11 | 0.049 | 0.650 |
| >3–5 | 31 |  |  |
| >5–7 | 22 |  |  |
| > 7 | 25 |  |  |
| N/A | 1 |  |  |
| **Vascular Invasion** | | | |
| Absent | 73 | 0.067 | 0.532 |
| Present | 17 |  |  |
| **Lymphatic Invasion** | | | |
| Absent | 26 | **0.215** | **0.041*** |
| Present | 64 |  |  |
| **Number of Positive Lymph Nodes** | | | |
| 0 | 26 | 0.206 | 0.052 |
| >1–3 | 20 |  |  |
| ≥ 4 | 44 |  |  |
| **T stage** | | | |
| T1 | 2 | 0.143 | 0.178 |
| T2 | 7 |  |  |
| T3 | 60 |  |  |
| T4 | 21 |  |  |
| **N stage** | | | |
| N0 | 26 | **0.222** | **0.035*** |
| N1 | 10 |  |  |
| N2 | 24 |  |  |
| N3 | 30 |  |  |
| **M stage** | | | |
| M0 | 89 | -0.031 | 0.772 |
| M1 | 1 |  |  |
| **WHO Type** | | | |
| Adenocarcinoma | 61 | -0.043 | 0.684 |
| Signet-ring Cell | 13 |  |  |
| Mucinous | 5 |  |  |
| Undifferentiated | 11 |  |  |
| **Borrmann Type** | | | |
| I | 10 | 0.019 | 0.861 |
| II | 20 |  |  |
| III | 52 |  |  |
| IV | 4 |  |  |
| N/A | 4 |  |  |
| **PD-L1^+^, %** | | | |
| ≤ 5 | 46 | -0.030 | 0.782 |
| >5–10 | 11 |  |  |
| >10–20 | 11 |  |  |
| > 20 | 20 |  |  |
| N/A | 2 |  |  |
| **CD8^+^, %** | | | |
| ≤ 5 | 41 | 0.054 | 0.617 |
| >5–10 | 22 |  |  |
| >10–20 | 17 |  |  |
| > 20 | 8 |  |  |
| N/A | 2 |  |  |
| **Survival time (Month)** | | | |
| ≤ 12 | 28 | **-0.254** | **0.029*** |
| >12–36 | 22 |  |  |
| >36–**60** | 6 |  |  |
| > 60 | 34 |  |  |

**P* < 0.05

**Table S2 Clinical characteristics of gastric cancer patients undergoing serum exosomal TAGLN2 detection (*n* = 30)**

| No. | Gender | Age | Borrmann Type | Grade | TNM | Tumor size | LVI | PNI | LNM | Distant Metastasis |
| --- | --- | --- | --- | --- | --- | --- | --- | --- | --- | --- |
| 1 | M | 72 | Ulcerative | G2 | pT1bN0M0 | 2.5×1.8×0.8 | N | Y | N | N |
| 2 | M | 74 | Protruded | － | pT4aN0M0 | 6.8×3.1×1.9 | N | Y | N | N |
| 3 | F | 72 | Ulcerative | G3 | pT4aN3aM0 | 8.5×6.7×2.4 | Y | Y | Y | N |
| 4 | M | 54 | Ulcerative | G3 | T4bN3aMx | 7.0×5.8×3.1 3.9×3.4×1.9 | Y | Y | Y | Y |
| 5 | F | 62 | Ulcerative | G3 | pT4bN3bM1 | 10.5×8.1×1.1 | Y | Y | Y | Y |
| 6 | F | 65 | Protruded | G3 | pT3N2M0 | 6.5×4.3×1.8 | Y | Y | Y | N |
| 7 | F | 70 | Ulcerative | G3 | T4aN1M0 | 4.3×3.3×1.1 | Y | Y | Y | N |
| 8 | F | 63 | Ulcerative | G3 | pT4aN3bM0 | 7.5×7.0×1.0 | Y | Y | Y | N |
| 9 | F | 51 | Infiltrative | G3 | － | － | － | － | － | Y |
| 10 | M | 62 | － | G3 | － | － | － | － | － | Y |
| 11 | M | 60 | － | G2 | cT4aNxMx | － | － | － | － | Y |
| 12 | F | 62 | Ulcerative | G3 | pT4bN1M0 | 3.2×2.2×0.8 | Y | Y | Y | N |
| 13 | M | 79 | Ulcerative | G2 | pT2N0M0 | 1.8×1.8×0.6 | Y | N | N | N |
| 14 | M | 78 | － | － | － | － | － | － | － | Y |
| 15 | F | 58 | － | － | － | － | － | － | － | Y |
| 16 | M | 71 | Ulcerative | G3 | pT4aN3bM0 | 7.5×5.5×1.1 | Y | Y | Y | N |
| 17 | M | 52 | Protruded | G2 | pT1bN0M0 | 5.4×4.3×0.6 2.7×2.1 | N | N | N | N |
| 18 | F | 54 | Ulcerative | G2 | pT3N2M0 | 5.6×4.3×1.0 | Y | Y | Y | N |
| 19 | M | 71 | － | G3 | cTxN1Mx | － | － | － | － | Y |
| 20 | F | 62 | － | G3 | cT4aNxM1 | － | － | － | － | Y |
| 21 | M | 70 | Ulcerative | G2 | pT4aN0Mx | 2.7×2.4×2.0 | Y | Y | N | N |
| 22 | M | 72 | Ulcerative | G2 | T4aN0M0 | 5.2×4.7×1.1 | Y | Y | N | N |
| 23 | M | 80 | － | － | cT4N2M1 | － | － | － | － | Y |
| 24 | M | 74 | Ulcerative | G3 | pT4bN3aM0 | 4.9×3.0×2.7 | Y | Y | Y | Y |
| 25 | F | 52 | － | G3 | － | － | － | － | － | Y |
| 26 | M | 52 | Protruded | G2 | pT1bN0M0 | 1.5×0.8 | N | N | N | N |
| 27 | M | 57 | Ulcerative | G2 | pT3N1M0 | 4.5×3.2×1.1 | Y | Y | Y | N |
| 28 | M | 55 | Ulcerative | － | pT4aN1M0 | 2.5×1.6×1.3 | Y | Y | Y | N |
| 29 | M | 56 | Ulcerative | G3 | cT4bN2M1 | 10.2×8.2×2.5 | Y | Y | Y | Y |
| 30 | M | 50 | Ulcerative | G3 | pT3N3aM0 | 2.3×1.6×0.6 | Y | Y | Y | N |

**Table S3 List of primers sequences for plasmids construction**

| **Gene** | **Foward Primer 5’—3’** | **Revers Primer 5’—3’** |
| --- | --- | --- |
| *TAGLN2* | CCCAAGCTTGCCACCATGGCCAACAGGGGACCTGC | ATAAGAATGCGGCCGCTCAGAGGATCTGGCGTGGCATCCCGTAG |
| *NRP1* | ATGGAGAGGGGGCTGCCGCTCCTCTGC | TTATTTGATACCTGATTGTATGGTGCTG |
| *SEMA4D* | ATGAGGATGTGCACCCCCATTAGGGGGC | TCAGTCTCCATCTGCGTCTGAGTCAGCGAA |
| *C/EBPβ* | ATGGAAGTGGCCGGTTTTTACG | TCAGCACTGGCCGGTGGCGGAG |
| *SP1* | ATGTACCCATACGACGTCCCAGACTACGCTAGCGACCAAGATCACTCCAT | TCAGAAGCCATTGCCACTGATATTAATGGA |
| *c-Jun* | ATGACTGCAAAGATGGAAACGACC | CCGCTCGAGTCAAAATGTTTGCAACTGCTGCG |
| *NRP1* ChIP1 | GAGGGAGTTAGTGGCTCAAATG | TAGGAGGTGCTGCAGAAATAAG |
| *NRP1* ChIP2 | TGCCAGGTCCTGAGAGGCGC | TCGCCGCCTCCCCAGGTGCAG |
| *NRP1* ChIP3 | CTTGGCGACTGGGAATCCCG | CAGGCCAGGACTGCGCGCTC |

**Table S4 List of siRNA target sequences for gene knockdown**

| **Gene** | **Sense Sequence 5’-3’** | **Antisense** |
| --- | --- | --- |
| si*TAGLN2-1* | CCAACUGGUUCCCUAAGAA | UUCUUAGGGAACCAGUUGG |
| si*TAGLN2-2* | CCAACUGGCCUCUUCCUUU | AAAGGAAGAGGCCAGUUGG |
| si*NRP1* | CUGAAUGUUCCCAGAACUA | UAGUUCUGGGAACAUUCAG |
| si*SEMA4D* | CGAACCAAAGAUCGUCAUCAA | UUGAUGACGAUCUUUGGUUCG |
| si*SP1* | GCUGGUGGUGAUGGAAUACAU | AUGUAUUCCAUCACCACCAGC |
| si*c-Jun* | CGGACCUUAUGGCUACAGUAA | UUACUGUAGCCAUAAGGUCCG |
| si*PlexinB1* | GUUCGACGUGCAAACAUCU | AGAUGUUUGCACGUCGAAC |

**Table S5 Primary antibodies used for western blot**

| **No.** | **Target Antigen** | **Vendor** | **Catalog Number** | **Purpose / Pathway** |
| --- | --- | --- | --- | --- |
| 1 | TAGLN2 | Proteintech | 15508-1-AP | Core protein of axis |
| 2 | NRP1 | R&D Systems | AF3870 | Core protein of axis |
| 3 | SEMA4D | R&D Systems | MAB74701 | Core protein of axis |
| 4 | VEGFR2 | R&D Systems | MAB3571-100 | Endothelial cell marker / Angiogenesis |
| 5 | CD31 | Santa Cruz Biotechnology | sc-376764 | Endothelial cell marker / Angiogenesis |
| 6 | vWF | Proteintech | 27186-1-AP | Endothelial cell marker / Angiogenesis |
| 7 | Cleaved Caspase 3 | Abcam | ab32042 | Apoptosis marker |
| 8 | Caspase 3 | Abcam | ab32351 | Apoptosis marker |
| 9 | Cleaved PARP1 | Abcam | ab32064 | Apoptosis marker |
| 10 | PARP1 | Abcam | ab191217 | Apoptosis marker |
| 11 | VE-cadherin | Santa Cruz Biotechnology | sc-9989 | Endothelial adherens junction protein |
| 12 | Occludin | Proteintech | 66378-1-Ig | Tight junction protein |
| 13 | ZO-1 | Proteintech | 21773-1-AP | Tight junction scaffolding protein |
| 14 | Claudin 5 | Abcam | ab15106 | Tight junction protein |
| 15 | Claudin 1 | Proteintech | 13050-1-AP | Tight junction protein |
| 16 | YAP | Proteintech | 66900-1-Ig | Hippo pathway |
| 17 | p-YAP (Ser127) | Abcam | ab76252 | Hippo pathway |
| 18 | MST1/2 | Affinity Biosciences | DF8569 | Hippo pathway |
| 19 | p-MST1/2 (Thr183/180) | Proteintech | 80093-1-RR | Hippo pathway |
| 20 | LATS1 | Proteintech | 66569-1-Ig | Hippo pathway |
| 21 | p-LATS1 (Thr1079) | Proteintech | 28998-1-AP | Hippo pathway |
| 22 | NF2 | Proteintech | 21686-1-AP | Hippo pathway |
| 23 | Ajuba | Proteintech | 28686-1-AP | Hippo pathway |
| 24 | PTPN14 | Proteintech | 67744-1-Ig | Hippo pathway |
| 25 | TAZ | Abcam | ab307440 | YAP transcriptional coactivator |
| 26 | β-Catenin | R&D Systems | MAB13291 | YAP/TAZ interactor |
| 27 | c-Jun | Abcam | ab40766 | Transcription factor, NRP1 regulator |
| 28 | SP1 | Proteintech | 66508-1-Ig | Transcription factor, NRP1 regulator |
| 29 | ERK1/2 | Abcam | ab17942 | MAPK signaling control |
| 30 | p-ERK1/2 | Abcam | ab278538 | MAPK signaling |
| 31 | E-cadherin | Abcam | ab231303 | EndoMT |
| 32 | N-cadherin | R&D Systems | AF6426 | EndoMT |
| 33 | Tie1 | Proteintech | 19329-1-AP | EndoMT |
| 34 | Tie2 | Proteintech | 19157-1-AP | EndoMT |
| 35 | α-SMA | Proteintech | 14395-1-AP | EndoMT |
| 36 | FSP-1 | MedChemExpress | HY-P84220 | EndoMT |
| 37 | Vimentin | Cell Signaling Technology | #5741 | EndoMT |
| 38 | Snail | Proteintech | 61367 | EndoMT |
| 39 | Slug | Proteintech | 12129-1-AP | EndoMT |
| 40 | Twist1 | abcam | ab323385 | EndoMT |
| 41 | ZEB1 | Cell Signaling Technology | #70512 | EndoMT |
| 42 | PlexinB1 | Proteintech | 23795-1-AP | RhoA/ROCK pathway |
| 43 | p-MLC2 (Thr18/Ser19) | Proteintech | 85804-1-RR | RhoA/ROCK pathway |
| 44 | p-MYPT1 (Thr696) | Cell Signaling Technology | #5 163 | RhoA/ROCK pathway |
